# An Integrated Strategy for Global Qualitative and Quantitative Profiling of Traditional Chinese Medicine Formulas: *Baoyuan* Decoction as a Case

**DOI:** 10.1038/srep38379

**Published:** 2016-12-07

**Authors:** Xiaoli Ma, Xiaoyu Guo, Yuelin Song, Lirui Qiao, Wenguang Wang, Mingbo Zhao, Pengfei Tu, Yong Jiang

**Affiliations:** 1 State Key Laboratory of Natural and Biomimetic Drugs, School of Pharmaceutical Sciences, Peking University, Beijing 100191, People’s Republic of China; 2 Modern Research Center for Traditional Chinese Medicine, Beijing University of Chinese Medicine, Beijing 100029, People’s Republic of China; 3 Waters corporation Shanghai Science & Technology Co Ltd, Shanghai 201206, People’s Republic of China

## Abstract

Clarification of the chemical composition of traditional Chinese medicine formulas (TCMFs) is a challenge due to the variety of structures and the complexity of plant matrices. Herein, an integrated strategy was developed by hyphenating ultra-performance liquid chromatography (UPLC), quadrupole time-of-flight (Q-TOF), hybrid triple quadrupole-linear ion trap mass spectrometry (Qtrap-MS), and the novel post-acquisition data processing software UNIFI to achieve automatic, rapid, accurate, and comprehensive qualitative and quantitative analysis of the chemical components in TCMFs. As a proof-of-concept, the chemical profiling of *Baoyuan* decoction (BYD), which is an ancient TCMF that is clinically used for the treatment of coronary heart disease that consists of Ginseng Radix et Rhizoma, Astragali Radix, Glycyrrhizae Radix et Rhizoma Praeparata Cum Melle, and Cinnamomi Cortex, was performed. As many as 236 compounds were plausibly or unambiguously identified, and 175 compounds were quantified or relatively quantified by the scheduled multiple reaction monitoring (sMRM) method. The findings demonstrate that the strategy integrating the rapidity of UNIFI software, the efficiency of UPLC, the accuracy of Q-TOF-MS, and the sensitivity and quantitation ability of Qtrap-MS provides a method for the efficient and comprehensive chemome characterization and quality control of complex TCMFs.

The clinical application and research of traditional Chinese medicine formulas (TCMFs) have drawn increasing attention in recent years because of their promising efficacies and minimal side effects, in particularly for multifactorial disorders^1^. Although well-accepted and widely used in China, TCMFs are considered as complementary and alternative medicines in many Western countries, mainly due to their complex chemical compositions, unclear effective material basis and action mechanisms, and unstable quality. Hence, more effort should be devoted to in-depth characterization of the chemome of TCMFs to interpret their clinical effects and to establish a comprehensive quality control method to ensure their stable clinical efficacy.

Ultra-performance liquid chromatography (UPLC) coupled with tandem mass spectrometry (MS/MS), *e.g.*, quadrupole-time of flight MS (Q-TOF-MS) or hybrid triple quadrupole-linear ion trap MS (Qtrap-MS), has been a work horse for the measurement of complex TCMFs because of its superiority in terms of separation efficiency, detection sensitivity, and structural characterization potency^2^,^3^. Q-TOF-MS has been shown to be intrinsically capable of comprehensively acquiring accurate mass spectral data based on MS^1^ full scan and MS^E^-based (also known as MS^All^) data-independent acquisition (DIA), indicating promising potential for the global chemical profiling of complex matrices^4^,^5^,^6^. However, once obtained, it is a complicated task to completely assign the huge dataset yielded from Q-TOF-MS, and it is even more challenging to exactly assign the fragment ion species generated by MS^E^ to their precursors for the co-eluting components, resulting in a significant barrier for structural identification. Although several post-acquisition data processing approaches, such as the mass defect filter (MDF) technique^7^ and diagnostic fragment ion (DFI)-based extension strategy^5^, have been developed to simplify the data processing and to increase the identification confidence, it is still formidable and labor-intensive to achieve the systematic chemical profiling of a complex TCMF.

In response to the shortcomings of the data acquisition and post-acquisition procedures of Q-TOF-MS, information-dependent acquisition (IDA, also known as data-dependent acquisition) of Qtrap-MS and UNIFI, a versatile and automated data processing platform, are adopted in the current study. Qtrap-MS has been widely demonstrated as a powerful apparatus due to its unique ability of simultaneous qualitative and quantitative measurement, usually in an information-dependent manner^8^,^9^. Compared with Q-TOF-MS, Qtrap-MS can dramatically increase the data quality, despite its low resolution, by utilizing prior knowledge-based acquisition modes, for instance, the precursor ion (Prec) and predictive multiple reaction monitoring (pMRM) modes. Survey experiments can trigger an enhanced product ion (EPI) scan *via* IDA method to acquire MS/MS data for the selected precursor ions. Moreover, Qtrap-MS can also perform the simultaneous quantitation of numerous analytes with largely different concentrations in complex samples using the scheduled MRM (sMRM) mode without compromising data quality *via* automatic alteration of the dwell time to maintain the desired cycle time^10^,^11^. Therefore, Qtrap-MS can act as a complementary qualitative and quantitative tool for Q-TOF-MS. Developed from the core-idea of database searching, the fully automated UNIFI software can accomplish chromatographic peak detection, molecular formula prediction, TCM database retrieval, MS/MS fragment matching, and preliminary chemical characterization almost without human assistance, suggesting that this software dramatically alleviates the workload for mining chemical structures from massive Q-TOF-MS datasets. Several applications of UNIFI have been published^12^,^13^,^14^,^15^, and the feasibility of UNIFI for chemical profiling of TCMFs, which is an extremely complicated compound pool, has not been systematically proved.

*Baoyuan* decoction (BYD), a well-known TCMF for original Qi vacuity, was initially archived in *Bo Ai Xin Jian* in the Ming dynasty. In modern clinical applications, BYD is a famous TCMF for the treatment of coronary heart disease, aplastic anemia, and chronic renal failure^16^,^17^. BYD consists of four famous herbal drugs, i.e., Ginseng Radix et Rhizoma (Chinese name: *Renshen*), Astragali Radix (*Huangqi*), Glycyrrhizae Radix et Rhizoma Praeparata Cum Melle (*Zhigancao*), and Cinnamomi Cortex (*Rougui*). However, the chemical profile of BYD has been scarcely reported, and only 30 flavonoids have been isolated and identified from BYD^18^,^19^, in contrast to its well-defined pharmacological patterns and clinical benefits.

As a consequence, we aim to propose a systematic strategy by integrating all the merits of UPLC, Q-TOF-MS, Qtrap-MS, and UNIFI software for the rapid and comprehensive qualitative and quantitative characterization of the chemome of BYD. The strategy consists of three steps as illustrated in Fig. 1. The first step is to search for and characterize the primary components of BYD by Q-TOF-MS, in which UNIFI software is used for automated processing of the dataset acquired by the MS^E^ scan mode with the assistance of an in-house library containing all the mass spectrometric information of BYD archived in the literature. The second step is to mine and identify the minor and trace components, for which IDA on UPLC/Qtrap-MS and DIA on UPLC/Q-TOF-MS were performed, in combination with mass fragmentation pathway analyses. Finally, almost all the detected compounds were quantified or relatively quantified by the sMRM mode on a Qtrap-MS. A total of 236 compounds were identified, including 139 saponins, 83 flavonoids, 6 procyanidins, 4 lignans, and 4 diterpenes. Thirty-six representative components were accurately quantified, and 139 components were relatively quantified. These findings provide a facile and practical tool for the rapid and comprehensive qualitative and quantitative profiling and quality control of BYD.

## Results

### Fragmentation rules and DFIs of saponins and flavonoids

Saponins and flavonoids have been identified as the dominant chemical homologues in Ginseng Radix et Rhizoma, Astragali Radix, and Glycyrrhizae Radix et Rhizoma, and thereby serve as the primary chemical classes in BYD. Because attention has been given to the mass fragmentation pathways of ginsenosides, astragalosides, licorice saponins, and flavonoids^20^,^21^,^22^,^23^,^24^,^25^, the applicability of those cracking rules archived in the literature were verified in this study by employing several representatives, including nine ginsenosides, four astragalosides, ten licorice saponins, and five flavonoids. Moreover, due to the great convenience provided by DFI filtering^5^ for compound searching and chemical identification, these authentic compounds were also employed to summarize the DFIs for the compounds with the above four chemical categories.

Nine ginsenosides, including protopanaxadiol (PPD)-type (*e.g.*, ginsenosides Rb1, Rb3, Rd), protopanaxatriol (PPT)-type (*e.g.*, ginsenosides Re, Rg1, Rg2), and other rare aglycone skeleton types (*e.g.*, ginsenosides Ro, Rg5, Rk1)^24^ share similar tandem spectral profiles, and prominent signals were observed as formic acid adduct ions ([M + HCOO]^−^) and deprotonated aglycone ions ([A−H]^−^) yielded by successive cleavage of sugar residues. Taking ginsenoside Re (PPT-type) as an example, significant signals at *m/z* 991.550 and 945.545 (see Fig. S1) were assigned to the formic acid adduct ion and the deprotonated molecular ion, respectively, and the DFI for the PPT derivatives was generated at *m/z* 475.379 by successive cleavage of two glucosyl (162 u) and one rhamnosyl (146 u) residues (see Supplementary Fig. S2A).

Similar to ginsenosides, [M + HCOO]^−^, [M−H]^−^, and [A−H]^−^ ions were afforded as dominant signals in the mass spectral profiles of all four astragalosides. Hence, successive neutral losses of sugar residues and acetyl moieties (if applicable) dominated the fragmentation pathways of astragalosides (see Supplementary Fig. S2B).

Most saponins from licorice are oleanane-type triterpene saponins (OTSs), *e.g.*, glycyrrhizic acid, licorice-saponins E2, G2, J2, H2, B2, and A3, uralsaponins C and F, and 22*β*-acetoxyglycyrrhizin. Unlike ginsenosides, the deprotonated molecular ions ([M−H]^−^) and the deprotonated ion of the diglucuronic acid residue (*m/z* 351.057, B_2_^−^) were prominent signals for the OTSs (see Supplementary Fig. S2C), whereas formic acid adduct ions were rarely detected, and Y_0_^−^ and Y_1_^−^ ions were occasionally observed^22^.

Five representative flavonoids, liquiritin apioside, isoliquiritin apioside, calycosin-7-*O*-*β*-d-glucopyranoside, (3 *R*)-(+)-isomucronulatol-2′-*O*-*β*-d-glucopyranoside, and apigenin-6,8-di-*C*-*β*-d-glucopyranoside were used to analyze the mass spectral properties of flavonoids. Similar behaviors were observed for the four flavonoid *O*-glycosides, such as formic acid adduct ions ([M + HCOO]^−^) and deprotonated ions ([M−H]^−^), deprotonated aglycone ions ([A−H]^−^), and some fragments yielded from retro Diels-Alder (RDA) reactions of aglycones^23^ (see Supplementary Fig. S4A–D). For instance, liquiritin gave a deprotonated ion at *m/z* 417.119 [M−H]^−^ in the MS spectrum, and a characteristic aglycone ion at *m/z* 255.066 through neutral loss of one glucosyl residue (162u) and two abundant fragment ions at *m/z* 135.016 (^1,3^A^−^) and 119.058 (^1,3^B^−^) generated from RDA reaction in the MS/MS spectrum (see Supplementary Fig. S3). The [A−H]^−^ ions were absent for the flavonoid *C*-glycosides due to the stable C-C bond between the aglycone and sugar residue. Instead, neutral losses of (CH_2_O)_n_ (*n* = 2–4) *via* cross-ring cleavage was the fragmentation behavior^23^. Taking apigenin-6,8-di-*C*-*β*-d-glucopyranoside as an example, significant distribution occurred for the fragments at *m/z* 473.109 (^0,2^ X _0α_^−^), 383.077 (^0,3^ X _0α_∙^0,2^ X _0β_^−^/^0,2^ X _0α_∙^0,3^ X _0β_^−^), and 353.067 (^0,2^ X _0α_∙^0,2^ X _0β_^−^)^26^ corresponding to successive neutral losses of 120.042 u (593.151 → 473.109 or 473.109 → 353.067) and 90.032 u (473.109 → 383.077) in the MS/MS spectrum (see Supplementary Fig. S4E).

A total of 389 compounds, mainly saponins and flavonoids, have been reported from the four single herbs of BYD, and all of them were included to construct an in-house library. DFIs were proposed for all chemical subtypes based on the fragmentation described above (see Supplementary Fig. S5). For ginsenosides, some other DFIs found in the literature, such as *m/z* 441.374^27^, 455.353^28^, 457.369^29^, 477.395^30^, 491.374^31^, 493.390^30^, and 507.369^32^ corresponding to the various aglycones of ginsenosides (see Supplementary Fig. S5), were included in the ginsenoside-focused screening, in addition to the well-defined DFIs of *m/z* 459.384 and *m/z* 475.379 for PPD- and PPT-type ginsenosides, respectively^25^. The [A−H]^−^ aglycone ion at *m/z* 489.359 was used as the DFI for searching astragalosides (see Supplementary Fig. S5). Moreover, the ion at *m/z* 351.057 (B_2_^−^) served as the DFI for mining licorice OTSs because diglucuronic acid substitution occurred for most licorice saponins. For flavonoids that primarily originated from Astragali Radix and Glycyrrhiza Radix, various characteristic aglycone ions such as *m/z* 253.051, 255.066, 267.066, 269.046, 269.081, 271.061, 283.061, 285.061, 289.071, 299.056, 299.093, and 301.110, were used to screen the flavonoids (see Supplementary Fig. S5), and the neutral losses of 120 u and 90 u served as the diagnostic cleavages for flavonoid *C*-glycosides.

### Integrated strategy for the comprehensive chemical characterization of BYD

The ingredients in a given matrix can be broadly sub-divided into primary and minor components^2^. The primary components usually afford significant LC-MS response, whereas the minor components suffer from extensive co-elution with the primary components and insufficient sensitivity of the adopted method^33^. Currently, in-depth profiling the primary constituents is a laborious and time-consuming task, let alone the minor components. Therefore, a systematic strategy was proposed to rapidly and comprehensively screen the chemical constituents in BYD. Firstly, an in-house library that covers most primary components in BYD was constructed, and UNIFI software and Q-TOF-MS were combined to perform automated data mining and structural assignment of the primary constituents. Secondly, several sensitive IDA-mediated methods were applied to the Qtrap-MS domain to extract information belonging to the minor constituents when they were co-eluted with the primary ones, and the mass fragmentation pattern-assisted structural identification was performed by integrating the low-resolution and high-resolution mass spectral information obtained from Qtrap-MS and Q-TOF-MS, respectively.

### Automated identification of major components

**Figure 2:**
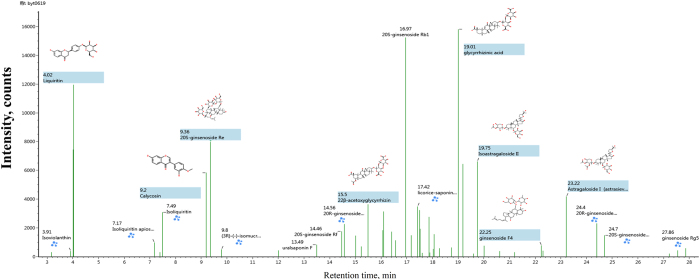
The sketch map of automated structural identification of components in *Baoyuan* decoction by UNIFI. The signals highlighted in blue are automatically identified according to MS data matching, whereas the signals tagged in blue automatically identified according to MS/MS data matching.

A versatile data process platform, UNIFI software, was used for the automated processing of the dataset acquired by MS^E^ mode of UPLC/Q-TOF-MS with the assistance of an in-house compound library. Because parameter setting plays a pivotal role in processing outcomes^12^, the parameters were carefully validated in terms of the accuracy and comprehensiveness of the detection results. The intensity threshold was set at 100cps as a compromise to improve the detection sensitivity while avoiding false positive detection. Regarding peak assignment, the candidate mass-to-charge ratios were automatically matched with the information recorded in the library *via* three important parameters, mass tolerance, adducts/pseudo-molecular ions, and fragment ions, and a candidate compound list was directly outputted. The wide mass tolerance could lead to a higher identification rate, resulting in a higher false detection rate. Thus, 5 ppm was set as a compromise based on the generally acceptable mass error range for accurate analysis. The deprotonated molecular ions ([M−H]^−^) together with the adduct ions ([M + HCOO]^−^ and [M + Cl]^−^) were automatically considered to enhance the selectivity (see Supplementary Fig. S1 and Fig. S3). Attention was also paid to the fragment ion mapping to estimate the rationality of the candidate compounds, which could allow the fragment ions to be automatically recognized and marked with blue tags in the MS/MS spectra (see Supplementary Fig. S1 and Fig. S3). The reliability and accuracy of UNIFI for the automated detection and identification of primary compounds in BYD were demonstrated by analyzing 49 authentic compounds, including 32 saponins, 14 flavonoids, and 3 diterpenes, that were isolated from BYD or its constituent herbs. All 49 reference compounds were rapidly and accurately captured by UNIFI. Following the UNIFI-mediated data processing, 113 compounds (see Fig. 2 and Table 1) were rapidly detected and identified, including 37 ginsenosides, 10 astragalosides, 24 licorice saponins, 28 flavonoids, 6 procyanidins, 4 lignans, and 4 diterpenes.

### Minor components characterization

The detection of minor components was performed by combining Qtrap-MS and Q-TOF-MS. Because comparable sensitivity has been demonstrated between these two analytical platforms^34^,^35^, most of the components found by various modes of Qtrap-MS were included in the dataset from Q-TOF-MS. Therefore, a workflow was designed to detect and identify the minor components, mainly saponins and flavonoids, in BYD using these two techniques. Firstly, the mass spectral information of the paired precursor-to-product ions afforded by Qtrap-MS guided the extraction of the corresponding ion information by Q-TOF-MS. Then, the proposed DFIs and fragmentation rules, in particular neutral loss (NL) and RDA reactions, were introduced for structural identification.

### Saponin- and flavonoid-focused compound screening by Qtrap-MS

As a complementary tool for Q-TOF-MS, Qtrap-MS is advantageous for highlighting the distribution of certain chemical homologues in complex matrices using some targeted screening methods. Based on the aforementioned mass fragmentation patterns of saponins and flavonoids, several survey experiments, such as Prec, pMRM, and MIM^36^,^37^, were applied to search for saponins and flavonoids in BYD, and an EPI scan was triggered to generate the MS/MS spectra.

The pMRM and MIM modes were combined to screen the saponins in BYD. The pMRM was carried out following a previously published procedure^38^ with a modified mass range of *m/z* 441–1255 for the Q1 cell. Because some saponins, such as licorice saponins, only exhibit [M−H]^−^ ions rather than adduct ions in their MS spectra, an MIM scan was introduced, with an identical mass range as pMRM. As a consequence, a total of 139 saponins were identified out and their MS and MS/MS spectral data are summarized in Table 1 and Table S1. All the saponins found with UNIFI were also detected using this method.

The flavonoids in BYD can be divided into aglycones, *O*-glycosides, and *C*-glycosides, and different scan modes were integrated to comprehensively detect the flavonoids. Firstly, a stepped MIM scan was used to record the potential flavonoid aglycones. The minimum molecular weight of the flavonoid skeleton is 222 u, and the molecular weight of a natural flavonoid should be at least 238 u due to the substitution of at least one hydroxy group^39^. Therefore, the mass range was set to *m/z* 237–401, corresponding to the substitution of at least one hydroxy group and at most six methoxy groups^39^. Consequently, signals at *m/z* 253, 255, 267, 269, 271, 283, 285, 289, 299, and 301 were revealed for the aglycones. Then, the aglycone ions were utilized to screen for flavonoid *O*-glycosides using Prec scan mode, and MIM mode from *m/z* 401–727 was used for flavonoid *C*-glycoside screening because *C*-glycosides contain at most two sugar substituents (2 × 162 u)^40^. In total, 12 flavonoid aglycones, 41 flavonoid *O*-glycosides, and 3 flavonoid *C*-glycosides were detected, and the fragment information obtained from EPI was carefully assigned to the corresponding precursor ions. Similar to the saponins, the flavonoids identified by UNIFI were also detected by Qtrap-MS.

### Structural identification of minor saponins

Following the introduction of the mass spectral information obtained from Qtrap-MS to the Q-TOF-MS dataset, accurate MS and MS/MS data were assigned to their corresponding compounds, and structural identification was performed. Among the detected saponins, 35 minor saponins were readily assigned as ginsenosides based on their observed aglycone ions, following the manual identification workflow (Table 1). Moreover, the successive neutral losses assisted in characterizing the glycan chain, such as cleavages of 162 u, 146 u, and 132 u corresponding to glucosyl, rhamnosyl, and arabinosyl or xylosyl residues, respectively. For example, S_PG_-17 was easily elucidated as a PPT-type ginsenoside from the observed [A−H]^−^ ion at *m/z* 475.379, and the fragment ions at *m/z* 799.485 and 637.432 corresponding to the neutral dissociations of one and two glucosyl residues; hence, it was identified as an isomer of Rg1. A total of 32 minor OTSs were rapidly classified and identified according to the summarized DFIs (Table 1). For instance, the [M−H]^−^ ion of S_GU_-13 was observed at *m/z* 895.396 corresponding to a molecular formula of C_44_H_64_O_19_. The dominant fragment ion at *m/z* 351.057 suggested that two glucuronosyl moieties exist in the structure of S_GU_-13. By matching with the in-house library, S_GU_-13 was tentatively deduced as an isomer of uralsaponin F. The characteristic ion at *m/z* 497.115 in the MS/MS spectra of S_GU_-16, S_GU_-20, and S_GU_-29 indicates the possible presence of a GlcA-GlcA-Rha chain in these OTSs^41^. Moreover, seven OTSs that contain single glucuronosyl moiety were also detected and putatively identified by the NL of 176.033 u.

### Structural identification of minor flavonoids

Based on our preliminary studies, the flavonoids in BYD can be structurally divided into seven sub-types (see Supplementary Fig. S5), including flavones, flavanones, isoflavones, chalcones, flavans, isoflavans, and pterocarpans. It was difficult to distinguish the aglycone skeleton types merely by the accurate mass data due to the wide occurrence of isomers. Thus, seven types of flavonoids were further sorted into four groups according to their specific fragmentation rules. Chalcones can be easily transformed to flavanones when encountering a high CE^39^ and produce the same fragment ions *via* RDA reaction, thus, flavanones and chalcones were classified into group I. The aglycone ions at *m/z* 255.066 (FA-1), 271.061 (FA-2), 269.081 (FA-6), and 285.061 (FA-8), corresponding to different substituents of the flavonoid aglycones, were utilized as DFIs for the detection of compounds in group I. Similarly, isoflavones and flavones afforded identical ^1,3^A^−^ and ^1,3^B^−^ ions; they were therefore assigned to group II. Several DFIs, such as FA-3 at *m/z* 253.051, FA-4 at *m/z* 267.066, FA-5 at *m/z* 269.046, FA-7 at *m/z* 283.061, and FA-10 at *m/z* 299.056 corresponding to diverse aglycones, might be generated by the compounds from group II. The flavans or isoflavans, which could produce [A−H]^−^ at *m/z* 289.071 (FA-10) and at *m/z* 301.110 (FA-8) were classified into group III. In addition, the pterocarpan derivatives, which were able to generate a characteristic [A−H]^−^ ion at *m/z* 299.0925 (FA-11), were defined as group IV.

Herein, the FA-1-related flavonoids were adopted to illustrate the structural characterization process. A total of 30 compounds were detected as liquiritigenin or isoliquiritigenin derivatives according to the prominent aglycone ion at *m/z* 255.066 and the ^1,3^A^−^ and ^1,3^B^−^ ions at *m/z* 135.016 and 119.058, respectively. Among them, six compounds were unambiguously verified by comparing with reference standards (F_GU_-30, 31, 59, 65, 66, and 83), whereas the identities of the other compounds were tentatively assigned by comparing with the data in the literature.

The sources of the components detected in BYD were proved by parallel measurement of the single herbal medicines (see Table 1 and Supplementary Fig. S6).

### Quantitative and semi-quantitative analysis of the detected components

**Figure 3:**
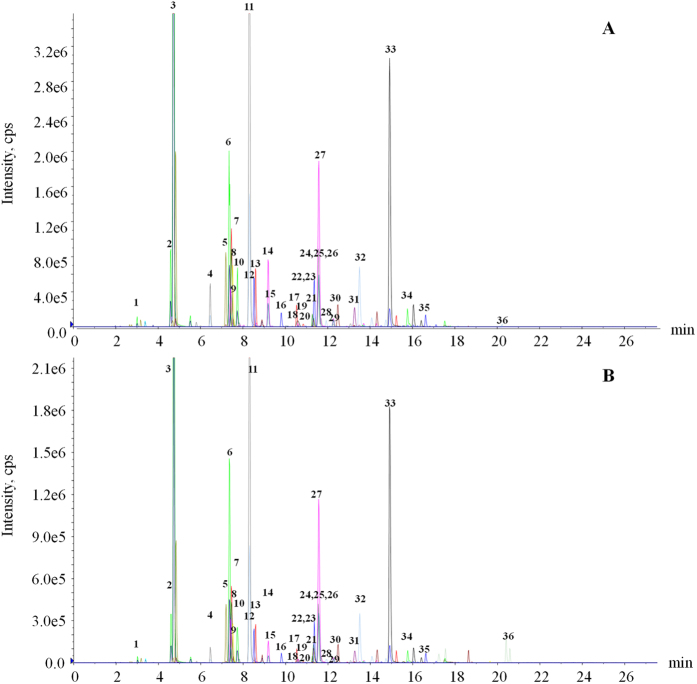
The extracted ion chromatograms (EICs) of references and BYD. (**A**) EIC of 36 ion transitions monitored under negative polarity for mixed references; (**B**) EIC of 36 ion transitions monitored under negative polarity for the BYD extract. The compound numbers are same as those described in Table 2.

sMRM mode is superior to the common MRM mode when a large number of ion transitions are involved in the quantitative analysis; thus, it was introduced in the present study to simultaneously monitor 175 compounds detected under the quantitation condition. The detailed parameters, including ion transitions, corresponding *t*_R_, and optimal DPs and CEs for the 175 targeted analytes are listed in Table S2. Based on the comprehensive semi-quantitative analysis of BYD by sMRM mode, 36 representative primary components of them, including 11 flavonoids and 25 saponins, were selected for simultaneous, absolute quantitative analysis. The representative chromatograms of BYD and the extracted ion chromatogram (EIC) of the mixed standards are shown in Fig. 3. All 36 analytes showed good linear regression (*r*^2^ > 0.999) within the test ranges. The LODs of the compounds were 0.04–23.21 ng/mL, and the LOQs were 0.17–55.25 ng/mL. These data are summarized in Table S3, indicating that sMRM is sensitive enough to quantitatively determine the large-scale analytes. The relative standard deviation (RSD) values of the intra- and inter-day precision studies were less than 5.23% (Table S4), indicating that the developed method exhibits satisfactory precision. The recoveries were between 90.68% and 108.92%, with RSDs less than 10%, which meets the quantitative criteria for multi-analytes in complex matrices. Satisfactory repeatability was demonstrated by RSDs of less than 5.01% for all the analytes, and the results of the stability assay suggested that the samples remained stable during measurement. The developed sMRM method was then applied to the simultaneous quantification of 36 analytes in six repeated batches of BYD extracts, and the data are shown in Table 2.

## Discussion

The efficacy and safety of TCMFs have been demonstrated by the long application history in China and other East Asian countries, such as Korea and Japan. It remains a great challenge to comprehensively understand the chemical composition of TCMFs, although great efforts have been devoted and some state-of-the-art analytical platforms, such as UPLC-Q-TOF-MS and UPLC-Qtrap-MS have been introduced. Attention has been given to the chemical fingerprinting of Ginseng Radix, Astragali Radix, Glycyrrhizae Radix, and Cinnamomi Cortex; however, the chemical composition of BYD has not been thoroughly studied because the decoction process might generate new chemical components through complex chemical reactions^42^. In addition, the contents of certain compounds cannot be calculated based on using the mixture ratio of single herbs in a TCMF because drug-drug interactions could occur during the decoction process^43^,^44^. For instance, the content of isoflavonoids and astragalosides in BYD was significantly greater than those in the single herbs based on direct comparison of the peak areas; some new compounds, such as ginsenosides S_PG_-1 and S_PG_-24, along with licorice saponins S_GU_-2, S_GU_-25, and S_GU_-39, were revealed in BYD, which are found in trace amounts in Ginseng Radix and Glycyrrhizae Radix but in higher amounts in BYD. Therefore, it is critical and necessary to characterize the chemical profile of TCMFs, even if the constituent herbs have been well defined because the chemical profile of a TCMF cannot be determined by simply pooling all the components from the single herbs.

Because it is a labor-intensive and time-consuming task to assess the quality of TCMFs by comprehensive characterization of their chemical profiles, it is usually feasible to conduct quality control of TCMFs by monitoring tens of components^45^,^46^. Although all components captured by UNIFI could be mined by manual data processing, the software has two attractive advantages compared with conventional chemical profiling workflows. First and foremost, the software is sufficiently versatile so that fully automated data processing can be achieved, and all candidate compounds are directly listed following the construction of an in-house library and the setting of the optimum parameters. Only 10 min is required for UNIFI to complete the analysis. Secondly, fragment ion matching can be adopted to improve the accuracy of UNIFI based on the predictive fragmentation pathways. For example, ginsenoside Re and liquiritin (see Supplementary Figs S1 and S3) were identified by matching not only their molecular ions but also the MS/MS fragment species with the information summarized in the library. Therefore, UNIFI provided a simple, efficient, and accurate method for primary component detection and identification of BYD, indicating a promising option for the chemical analysis and quality control of TCMFs, which are critical to ensure the efficacy and safety of TCMFs.

**Figure 4:**
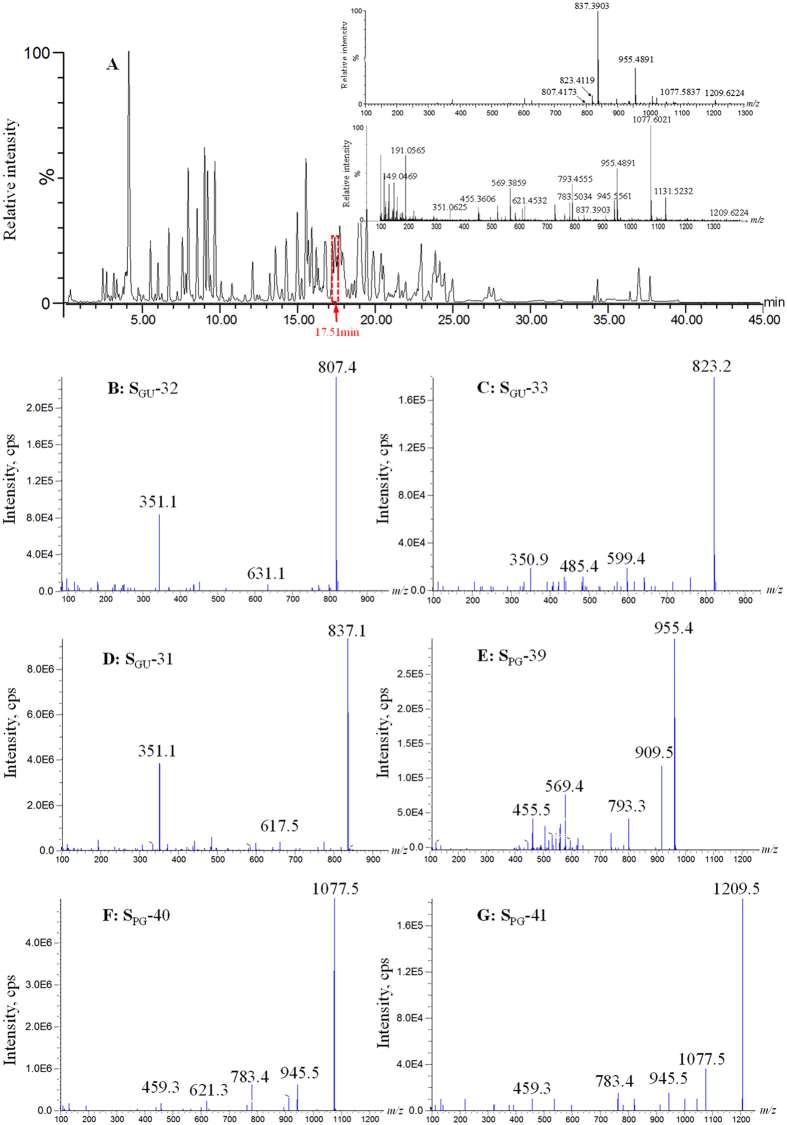
The base peak chromatogram (BPC) of *Banyuan* decoction extract and MS^E^ spectra at 17.51 min by UPLC/Q-TOF-MS (**A**); Enhanced product ions (EPI) spectra of co-eluting ions at *m/z* 807.4 (**B**), 823.4 (**C**), 837.4 (**D**), 955.5 (**E**), 1077.6 (**F**), and 1209.6 (**G**) using IDA analysis mode of UPLC/Qtrap-MS.

It may be as important to detect and identify the minor components as it is to detect and identify the primary components when attempting to comprehensively understand the chemical composition of TCMFs. In most cases, the characterization of the minor constituents suffers from their co-elution with the major components and from the insufficient sensitivity of the established method. Despite being useful for the detection and identification of primary compounds, UNIFI extensively neglects minor compounds. The MS and MS/MS signal intensities of the compounds in BYD span three orders of magnitude, resulting in the signals belonging to the minor components being easily submerged by their co-eluting primary components. Therefore, IDA using Qtrap-MS was introduced as a complementary method for MS^E^ by Q-TOF-MS to provide a deeper data mining of the MS^E^ dataset. The Q3 cell of the Qtrap-MS enables rapid switching between conventional radiofrequency/arc (RF/DC) resolving quadrupole mass filter to perform sensitive MRM or NL scanning and linear ion trap (LIT) apparatus to perform EPI scanning^47^. In particular, the scan rate of the LIT of 20000 Da/s could fulfill the demands of acquisition of high-quality MS/MS spectral data for all precursor ions that pass the IDA threshold. Although great sensitivity can be obtained by the MS^E^ mode in Q-TOF-MS, all co-eluted precursor ions simultaneously rush into the collision cell to generate fragment ion species which are further transmitted to the TOF chamber of the Q-TOF-MS at the same time due to the wide pass Q1 mode^48^. Therefore, the fragment species of all co-eluted compounds share a single MS/MS spectrum and the exact pairing of precursor ions with the corresponding product ion cannot be achieved. Alternatively, the narrow-pass Q1 mode is normally employed for IDA, which affords MS/MS spectra with minimal interference because only precursor ions meeting the preset criteria within the selected narrow *m/z* window (0.6–0.8 Da wide for unit resolution) are transferred to the collision cell to generate product ions. In other words, separate acquisition of the MS/MS spectra is theoretically guaranteed for all Q1 signals detected by pMRM/MIM/Prec mode. Therefore, IDA mode provides useful guidance for the assignment of accurate mass spectral data from sophisticated Q-TOF-MS chromatograms under MS^E^ mode. In the present study, the MIM and pMRM scanning methods were developed to screen potential saponins based on the mass spectrometric behavior obtained with the assistance of several authentic compounds, and the detection of 67 minor saponins, including 35 ginsenosides and 32 OTSs was achieved. MIM and Prec scanning modes were applied to systematically detect flavonoids because of the various DFIs for flavonoids in BYD, resulting in the observation of 56 additional minor flavonoids, including 12 flavonoid aglycones, 41 flavonoid *O*-glycosides, and 3 *C*-glycoside. Based on the stepped MIM/pMRM and MIM/Prec scans, the chromatographic peaks with co-elution phenomenon could be easily distinguished and extracted. For instance, a total of six MS^2^ spectra were obtained for co-eluted [M−H]^−^ ions of *m/z* 807, 823, 837, 955, 1077, and 1209, respectively, by Qtrap-MS at 17.51 min (see Fig. 4), whereas these fragment ions of *m/z* 807.417, 823.412, 837.390, 955.489, 1077.584, and 1209.622 co-existed in a single Q-TOF-MS spectrum. Following the accurate matching of fragment ions with their respective precursor ions in the high-resolution MS spectrum and analyzing all ion species using the proposed fragmentation patterns, the six co-eluted compounds were identified as yunganoside I2 or licorice saponin B2, uralsaponin P, uralsaponin U or uralsaponin N, ginsenoside Ro, ginsenoside Rc, and ginsenoside Ra1.

By combining the rapid and automated identification by UNIFI, the sensitive targeted detection by Qtrap-MS, and the accurate mass measurements by Q-TOF-MS, an LC-MS-based qualitative analysis strategy consisting of two progressive steps was proposed for the rapid, accurate, and global chemical profiling of BYD. The cracking rules and DFIs of the primary chemical homologues in BYD were proposed by employing several representatives, and the mass spectral patterns assisted the structural identification of flavonoids and saponins. For the first step, 113 major components were rapidly identified by automated data-processing with UNIFI software in approximately ten minutes. In particular, the identities of 49 components were confirmed by comparison with the reference standards. In the second step, as many as 123 minor compounds (mainly saponins and flavonoids) were systematically detected from BYD by multiple screening methods based on UPLC/Qtrap-MS and were putatively identified by cross-talking between Qtrap-MS and Q-TOF-MS. Twenty of the compounds were identified as possibly new compounds, including seven licorice saponins (S_GU_-2, S_GU_-5, S_GU_-14, S_GU_-25, S_GU_-27, S_GU_-39, S_GU_-52), seven ginsenosides (S_PG_-1, S_PG_-21, S_PG_-23, S_PG_-24, S_PG_-27, S_PG_-36, S_PG_-58), and six flavonoids (F_AM_-51, F_AM_-55, F_GU_-14, F_GU_-15, F_GU_-32, F_GU_-74). Altogether, 236 compounds were identified from BYD, including 139 saponins, 83 flavonoids, 6 procyanidins, 4 lignans, and 4 diterpenes. Furthermore, the quantitation of 36 primary compounds and the relative quantification of 139 compounds were performed by sMRM using Qtrap-MS for the quality control of BYD.

These findings systematically illustrated the comprehensive chemical composition of BYD and provided the valuable evidences for clarification of the therapeutic material basis and action mechanism of this formula. Saponins and flavonoids were disclosed to be the main components in BYD, and many of them have been reported to have a verity of pharmacological activities on cardiovascular system, which is the main clinical application of BYD. For example, ginsenosides, including Re, Rb1, and Rg1, have the ability to protect the myocardia against injury produced by ischemia and reperfusion^49^; astragaloside IV, one of the main active ingredients in Astragali Radix, has the functions of vasodilating effect and protecting the vascular endothelial cells^50^; calycosin has the protective action against cardiac injury^51^; glycyrrhizin was identified as a thrombin inhibitor *in vitro* and *in vivo*^52^; isoliquiritigenin and isoliquiritin, two main active flavonoids of licorice, were reported to have a vasorelaxant effect and be able to decrease the tube formation in vascular endothelial cells^53^,^54^.

In conclusion, the integrated LC-MS-based strategy provided a meaningful and practical workflow for the rapid, accurate, and comprehensive identification and quantitation of the complicated TCMFs, which will supply valuable references for the further interpretation of their clinical effects, action mechanism, and quality control.

## Methods

### Materials and reagents

All crude materials were collected from a TCM market (Anguo, Hebei, China). Their herbal origins were authenticated by one of the authors (P.F. Tu). Authentic saponins, including ginsenosides Rb1, Rb2, Rb3, Rc, Rd, Re, Rf, Rg1, Rg2, Rg3, Rg5, Rg6, Rh1, Rh2, Rk1, Ro, and F4, pseudoginsenoside F11, notoginsenoside R2, astragalosides II, III, and IV, isoastragaloside II, glycyrrhizic acid, and 18*β*-glycyrrhetic acid were supplied by Chengdu Must Bio-technology Co., Ltd. (Chengdu, Sichuan, China), whilst uralsaponins C and F, 22*β*-acetoxyglycyrrhizin, and licorice-saponins E2, G2, J2, H2, B2, and A3 were kindly provided by Prof. Min Ye (Peking University, Beijing, China)^55^. Reference flavonoids, such as (3 *R*)-(+)-isomucronulatol-2′-*O*-*β*-d-glucopyranoside, (3 *R*)-(−)-isomucronulatol-7-*O*-*β*-d-apiofuranosyl(1 → 2)-*β*-d-glucopyranoside, apigenin-6,8-di-*C*-*β*-d-glucopyranoside, neoisoliquiritin, calycosin, calycosin-7-*O*-*β*-d-glupyrancoside, liquiritigenin, liquiritin, isoliquiritigenin, isoliquiritin, isoliquiritin apioside, liquiritin apioside, licuraside, ononin, formononetin, and 7,4′-dihydroxyflavone were previously purified and identified from BYD in our laboratory^16^,^17^, whereas three diterpenes, i.e., anhydrocinnzeylanol, anhydrocinnzeylanine, and cinnzeylanine were purified and identified from Cinnamomi Cortex^56^. The purities of all the reference compounds were determined to be greater than 98% by UPLC-DAD.

Acetonitrile (ACN) and MeOH of LC-MS grade were obtained from Merck (Darmstadt, Germany). LC-MS grade formic acid was obtained from Sigma-Aldrich (Steinheim, Germany). Deionized water was prepared on a Millipore Milli-Q water purification system (Billerica, MA, USA).

### Sample preparation

Pulverized crude materials consisting of Ginseng Radix et Rhizoma (10 g), Astragali Radix (30 g), Glycyrrhizae Radix et Rhizoma Praeparata Cum Melle (10 g), and Cinnamomi Cortex (5 g) were immersed in 550 mL of deionized water for 1 h and were then heated under reflux for 1.5 h two times. Both extractants were combined, filtered, and freeze-dried into powder. Accurately weighed lyophilized powder (0.2 g) was thoroughly suspended in ten volumes of deionized water. After centrifugation at 9,600 rpm for 10 min, a 500 μL aliquot of the supernatant was loaded onto a preconditioned Phenomenex Strata-X SPE column (500 mg/5 mL, Torrance, CA, USA) and successively eluted with 6 mL of water and 6 mL of MeOH. The MeOH effluent was concentrated to dryness under reduced pressure, reconstituted in 1 mL of MeOH, and filtered through a 0.22-μm membrane prior to LC-MS analysis. The injection volume was 0.6 μL. Additionally, extract samples of the four constituent herbs of BYD were prepared in parallel.

A stock solution (1 mg/mL) of each reference sample was obtained by dissolving accurately weighed compound in MeOH. All solutions were maintained at −20 °C prior to use.

### UPLC/Q-TOF-MS conditions

The Waters ACQUITY UPLC system was connected to an Xevo-G2 Q-TOF mass spectrometer *via* an electrospray ionization (ESI) interface (Milford, MA, USA). Waters Empower software (Version 2) was used for apparatus synchronization and data acquisition and processing. Chromatographic separations were conducted on a Waters CORTECS UPLC C_18_ column (1.6 μm, 2.1 × 100 mm, Milford, MA, USA). The mobile phase consisting of 0.05% aqueous formic acid (A) and ACN containing 0.05% formic acid (B) was programmed in gradient as follows: 0.0–2.0 min, 2–15% B; 2.0–12.0 min, 15–25% B; 12.0–22.0 min, 25–40% B; 22.0–30.0 min, 40–60% B; 30.0–37.0 min, 60–100% B; 37.0–39.0 min, 100% B; 39.0–39.01 min, 100–2% B; and 39.01–45.0 min, 2% B. The flow rate was 0.4 mL/min. The temperatures of the column oven and auto-sampler were maintained at 35 °C and room temperature, respectively.

Leucine-enkephalin was used as the lock mass compound for accurate mass calibration. All MS^E^ data were acquired with negative polarity. The ion-source parameters were set as follows: source temperature, 110 °C; desolvation temperature, 450 °C; desolvation gas (N_2_), 650 L/h, and nebulizer gas, 20 L/h. Two separate runs were conducted using the optimal compound-dependent parameters for flavonoids and saponins. For the former, parameters were set as follows: the capillary voltage, −2.03 kV; cone voltage, −10 V; collision energy (CE), −6 eV for MS and −15 to −55 eV for MS/MS, respectively; and MS^1^ scan range, *m/z* 237–731. Regarding the latter, parameters were applied as follows: capillary voltage, −2.3 kV; cone voltage, −30 V; CE, −6 eV for MS and −30 to −70 eV for MS/MS, respectively; and MS^1^ scan range, *m/z* 441–1251.

Post-acquisition data processing was automatically performed by UNIFI software (v.1.6.0, Waters), and MassLynx 4.1 software (Waters) was utilized for minor compound identification. An in-house library that contained the molecular formulae, molecular weights, and chemical structures of 389 compounds that were previously isolated from Ginseng Radix et Rhizoma (75 compounds), Astragali Radix (109 compounds), Glycyrrhiza Radix et Rhizoma (162 compounds), and Cinnamomi Cortex (43 compounds) was constructed to assist the chemical identification. Moreover, the retention times and mass spectral information, particular the DFIs of the authentic compounds, were also included in the in-house library.

### UPLC/Qtrap-MS conditions for qualitative analysis

A Waters ACQUITY H-Class UPLC system (Milford, MA, USA) was connected online with an ABSciex 4500 Qtrap mass spectrometer (Foster City, CA, USA) *via* an ESI interface. The chromatographic separation program was identical to the method described above. An auto-sampler was responsible for triggering the mass spectrometer *via* a pulse signal.

In the mass domain, the ion source parameters were maintained as follows: polarity, negative; ion spray voltage, −4500 V; source temperature, 550 °C; curtain gas (CUR), 35 psi; ion source gas 1 (GS1), 55 psi; ion source gas 2 (GS2), 55 psi. The data acquisition and processing were performed by ABSciex Analyst 1.6.2 software. Some survey experiments, including pMRM mode, MIM mode, and Prec scan, were adopted to trigger EPI scans in the linear ion trap cell (scan rate, 20000 Da/s) through an IDA procedure to search for saponins and flavonoids.

The scanning programs for saponins were as follows:

#### pMRM-EPI

pMRM-EPI procedures in the literature were used with minor modifications^57^. Two separate runs were performed with the mass ranges of *m/z* 441.5–843.5 and *m/z* 843.5–1255.5 for Q1. The dwell time was set to 8 ms for each ion transition. The IDA threshold and the CE of EPI were set to 500 cps and −65 ± 20 eV, respectively.

#### MIM-EPI

Stepped MIM-EPI protocols developed in our previous study were implemented with minor modifications^58^. A total of 412 MIM transitions were fragmented into two separate runs from *m/z* 441.5–843.5 and *m/z* 843.5–1255.5 for Q1 with a step-size of 2 u. The dwell time was set to 8 ms for each transition. IDA threshold and CE of EPI were set at 500 cps and −65 ± 20 eV, respectively.

The scanning modes for flavonoids were as follows:

#### MIM-EPI

Two separate runs were performed at the mass range of *m/z* 237.5–401.5 and *m/z* 401.5–727.5 for Q1, with a step-size of 2 u. The dwell time was set to 8 ms for each transition. The IDA threshold and the CE of EPI were set to 500 cps and −35 ± 15 eV, respectively.

#### Prec-EPI

Prec scans of ions, including *m/z* 253, 255, 267, 269, 271, 283, 285, 289, 299, and 301, were performed using a fixed CE (−25 eV) in the scan range of *m/z* 237.5–727.5. The IDA threshold and CE of EPI were set to 2000 cps and −25 ± 15 eV, respectively.

### UPLC/Qtrap-MS conditions for quantitative analysis

The large-scale semi-quantitation of 139 compounds and simultaneous absolute quantitation of 36 representative compounds in BYD were performed on a UPLC/Qtrap-MS system using sMRM mode. To shorten the analysis time while maintaining satisfactory separation, the gradient elution program was optimized as follows: 0–1.0 min, 2–10% B; 1.0–8.5 min, 10–25% B; 8.5–10.0 min, 25–29.5% B; 10.0–12.0 min, 29.5–30% B; 12.0–16.0 min, 30–40% B; 16.0–20.0 min, 40–60% B; 20.0–23.5 min, 60–100% B; 23.5–24.5 min, 100–100% B; 24.5–24.55 min, 100–2% B; 24.55–27.5 min, 2–2% B. Six batches of BYD lyophilized powders were prepared using the procedure described above. The accurately weighed lyophilized powders of each batch (0.2 g) were thoroughly suspended in ten volumes of 5% aqueous ACN. After centrifugation at 9,600 rpm for 10 min, the supernatant was filtered through a 0.22 μm membrane prior to LC-MS analysis. The injection volume and other LC conditions were set as previously described.

Each of the 36 analytes was prepared at a concentration of approximately 100 ng/mL with 50% aqueous ACN. They were directly infused into the ESI interface to investigate their optimal mass spectrometric parameters, including DPs and CEs. The ion transitions, corresponding *t*_R_, and optimal DPs and CEs of the sMRM scan mode are shown in Table S3. The MRM detection window for each ion pair was set to 1.0 min, and the target scan time was set to 1.0s.

### Preparation of stock and working solutions

To improve the quantitation precision and repeatability, baicalin was chosen as the internal standard (IS) for the determination of 11 flavonoids, and tenuifolin was used as the IS for 25 saponins (Table S2).

### Calibration curves

A mixed solution containing all 36 references was diluted to the appropriate concentrations using 50% aqueous ACN to construct calibration curves. At least six concentrations of the solution were analyzed in duplicate, and then calibration curves were generated to confirm the linearity between the ratio of the peak areas (analyte/IS) and the concentrations of the 36 analytes.

### Assay validation of the scheduled MRM

The stock solutions were diluted to a series of appropriate concentrations with 50% aqueous methanol and were then injected into the LC-MS for analysis. The LODs and LOQs were determined as signal-to-noise ratios (S/N) of approximately 3 and 10, respectively. The repeatability of the method was determined by analyzing six replicates of a BYD sample and is represented as the relative standard deviation (RSD) of the content of each analyte. The intra- and inter-day variations were used to analyze the precision of the established method. For the intra-day variability test, six replicates of the same solution were analyzed on a single day, while for the inter-day variability test, the same solution was examined in triplicate on three consecutive days. The variations are expressed as the RSDs of the data. Stability tests were performed by analyzing the BYD sample solution over a period of 0 h, 2 h, 4 h, 8 h, 10 h, 12 h, and the RSD was used to evaluate the stability. Recovery tests were conducted on samples spiked with approximately 100% of known amounts of the analytes, with six replicates for each sample.

## Additional Information

**How to cite this article**: Ma, X. *et al*. An Integrated Strategy for Global Qualitative and Quantitative Profiling of Traditional Chinese Medicine Formulas: *Baoyuan* Decoction as a Case. *Sci. Rep.* **6**, 38379; doi: 10.1038/srep38379 (2016).

**Publisher's note:** Springer Nature remains neutral with regard to jurisdictional claims in published maps and institutional affiliations.

## Supplementary Material

Supplementary Information

## Figures and Tables

**Figure 1:**
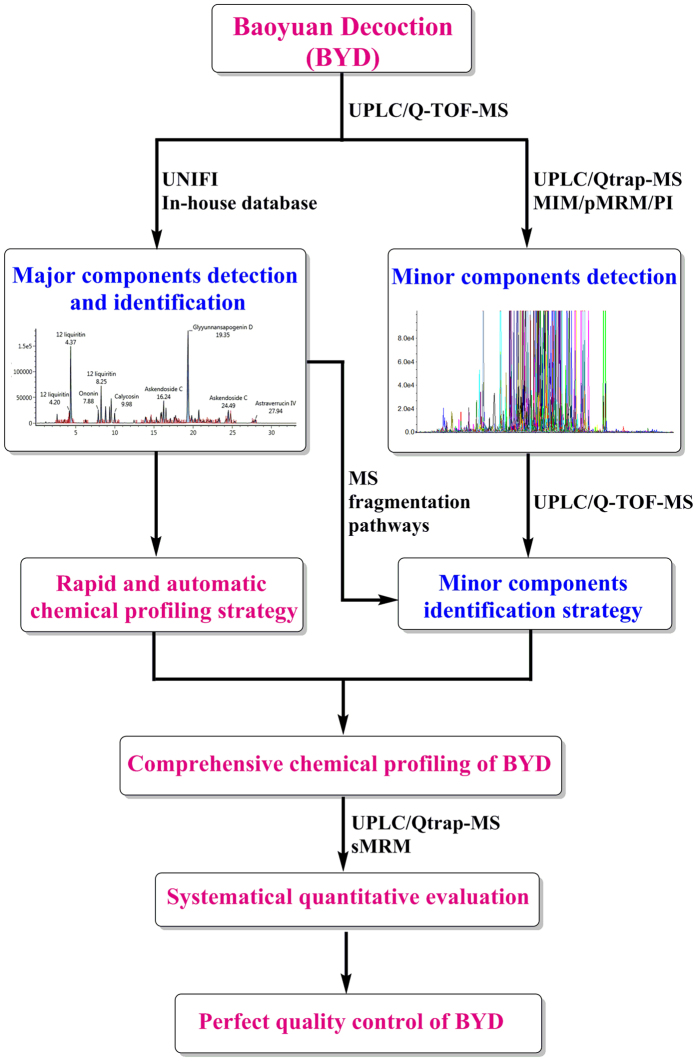
The workflow chart for global chemical profiling of *Baoyuan* decoction by integrated LC-MS strategy.

**Table 1:** Characterization of the chemical constituents of *Baoyuan* decoction by UPLC/Q-TOF-MS.

No.	*t*_R_ (min)	[M-H]^−^	Error	[M + COOH]^−^	Error	Formula	Identification^a^
S_GU_-1^m^	9.5	839.408	1.9			C_42_H_64_O_17_	Yunganoside G2
S_GU_-2^m^	10.94	969.4682	−1.3			C_48_H_74_O_20_	g-*O*-Rha-GlcA-GlcA
S_GU_-3*^C^	12.2	823.4131	1.8			C_42_H_64_O_16_	Uralsaponin C
S_GU_-4^m^	12.33	969.4682	−1.3			C_48_H_74_O_20_	Albiziasaponin B
S_GU_-5^m^	12.82	1027.4719	−3			C_50_H_76_O_22_	g-*O*-Acetyl-GlcA-GlcA-Glc
S_GU_-6^C^	12.97	835.3771	1.9			C_42_H_60_O_17_	24-Hydroxyl-licorice E2/Yunganoside M
S_GU_-7^m^	13.06	823.4142	3.2			C_42_H_64_O_16_	Uralsaponin C
S_GU_-8^m^	13.4	999.4434	−0.3			C_48_H_72_O_22_	24-Hydroxy-licorice-saponin A3
S_GU_-9*^C^	13.5	895.3962	−0.2			C_44_H_64_O_19_	Uralsaponin F
S_GU_-10	13.6	821.3938	−2.7			C_42_H_62_O_16_	Macedonoside C
S_GU_−11^C^	13.68	853.3877	2.2			C_42_H_62_O_18_	22-Hydroxy-licorice-saponin G2
S_GU_-12^m^	14.07	851.4052	−1.8			C_43_H_64_O_17_	Not identified
S_GU_-13^m^	14.13	895.397	0.7			C_44_H_64_O_19_	Isomer of uralsaponin F
S_GU_-14^m^	14.17	953.4739	−0.7			C_48_H_74_O_19_	d/e/f-*O*-Xyl(Ara)-GlcA-GlcA
S_GU_-15^C^	14.38	823.4111	−0.6			C_42_H_64_O_16_	Uralsaponin C
S_GU_-16^m^	14.6	1011.4815	1.4			C_49_H_74_O_19_	Licorice saponin D3
S_GU_-17^C^	15	835.3748	0.4			C_42_H_60_O_17_	24-Hydroxyl-licorice E2
S_GU_-18^C^	15.01	849.3528	−2.2			C_42_H_58_O_18_	Uralsapionin D
S_GU_-19^m^	15.16	835.375	−0.2			C_42_H_60_O_17_	Uralsaponin E
S_GU_-20^C^	15.22	1025.4581	−1.8			C_50_H_74_O_22_	Uralsaponin X
S_GU_-21*^C^	15.31	983.4489	0.1			C_48_H_72_O_21_	Licorice-saponin A3
S_GU_-22^m^	15.75	865.4229	0.8			C_44_H_66_O_17_	22*β*-Acetoxyglycyrrhizic acid
S_GU_-23*^C^	15.87	879.402	0.5			C_44_H_64_O_18_	22*β*-acetoxyglycyrrhizin
S_GU_-24^m^	16.03	837.3928	2.3			C_42_H_62_O_17_	Uralsaponin U/Uralsaponin N
S_GU_-25^m^	16.26	969.4703	0.8			C_48_H_74_O_20_	m/n-*O*-Xyl(Ara)-GlcA-GlcA
S_GU_-26^m^	16.3	865.4229	0.8			C_44_H_66_O_17_	22*β*-Acetoxy-licorice-saponin B2
S_GU_-27^m^	16.37	969.4703	0.8			C_48_H_74_O_20_	m/n-*O*-Xyl(Ara)-GlcA-GlcA
S_GU_-28^C^	16.53	823.4131	0.3			C_42_H_64_O_16_	Uralsaponin P
S_GU_-29^m^	16.83	953.4752	0.6			C_48_H_74_O_19_	Yunganoside H1
S_GU_-30*^C^	17.2	819.3803	0			C_42_H_60_O_16_	Licorice-saponin E2
S_GU_-31*^C^	17.41	837.3895	−1.7			C_42_H_62_O_17_	Licorice-saponin G2
S_GU_-32^m^	17.51	807.4173	0.7			C_42_H_64_O_15_	Yunganoside I2/Licorice saponin B2
S_GU_-33^C^	17.53	823.4119	0.4			C_42_H_64_O_16_	Uralsaponin P
S_GU_-34^m^	17.75	837.3932	2.7			C_42_H_62_O_17_	Uralsaponin U/Uralsaponin N
S_GU_-35^C^	17.78	819.3813	1.2			C_42_H_60_O_16_	Yunganoside E2
S_GU_-36^m^	18	863.4068	0.3			C_44_H_64_O_17_	22*β*-Acetoxyglycyrrhaldehyde
S_GU_-37^m^	18.12	953.4752	0.6			C_48_H_74_O_19_	Uralsaponin T
S_GU_-38^m^	18.3	879.402	0.5			C_44_H_64_O_18_	Uralsaponin M
S_GU_-39^m^	18.39	865.4229	0.4			C_42_H_62_O_17_	r-*O*-GlcA-Glc
S_GU_-40^m^	18.48	793.4029	2.4			C_41_H_62_O_15_	Not identified
S_GU_-41^m^	18.5	967.4534	−0.1			C_48_H_71_O_20_	Rhaoglycyrrhizin
S_GU_-42^C^	18.66	837.3912	0.4			C_42_H_62_O_17_	Uralsaponin U/Uralsaponin N
S_GU_-43^m^	18.75	865.4225	0.3			C_44_H_66_O_17_	22*β*-Acetoxy-licorice-saponin B2
S_GU_-44*^C^	19.01	821.4089	15.7			C_42_H_62_O_16_	Glycyrrhizic acid
S_GU_-45*^C^	19.11	821.4089	15.7			C_42_H_62_O_16_	Licorice-saponin H2
S_GU_-46^m^	19.23	807.4173	−4.5			C_42_H_64_O_15_	Glycyrflavoside C
S_GU_-47^C^	19.7	807.4169	0.2			C_42_H_64_O_15_	22-Dehydroxyl-uralsaponin C
S_GU_-48^C^	20.01	807.4164	−0.4			C_42_H_64_O_15_	Yunganoside I2
S_GU_-49^m^	20.14	807.4161	−0.7			C_42_H_64_O_15_	Yunganoside I2
S_GU_-50^m^	20.39	821.3961	0.1			C_42_H_62_O_16_	Licorice-saponin H2
S_GU_-51^C^	20.79	821.3961	0.1			C_42_H_62_O_16_	Isomer of licorice-saponin H2
S_GU_-52^m^	21.1	777.4047	−1.8			C_41_H_62_O_14_	d/e/f-*O*-GlcA-Glc
S_GU_-53*^C^	21.49	823.4103	−1.6			C_42_H_64_O_16_	Licorice saponin J2
S_GU_-54^C^	22.5	805.4028	2.2			C_42_H_62_O_15_	Uralsaponin W
S_GU_-55*^C^	22.98	807.4167	0			C_42_H_64_O_15_	Licorice-saponin B2
S_GU_-56*^m^	29.59	469.3318	0.4			C_30_H_46_O_4_	Glycyrrhetic acid
S_AM_-1^C^	15.02	945.5021	−4	991.5098	−1.6	C_47_H_78_O_19_	Astragaloside V
S_AM_-2^C^	16.8	945.5045	−1.5	991.5098	−1.6	C_47_H_78_O_19_	Astragaloside VI
S_AM_-3*^C^	17.9			829.458	−0.7	C_41_H_68_O_14_	Astragaloside III
S_AM_-4*^C^	18.1			829.458	−0.7	C_41_H_68_O_14_	Astragaloside IV
S_AM_-5*^C^	19.77			871.4691	0	C_43_H_70_O_15_	Astragaloside II
S_AM_-6^C^	20.89			871.4691	0	C_43_H_70_O_15_	Cyclogaleginoside D
S_AM_-7*^C^	21.92			871.4691	0	C_43_H_70_O_15_	Isoastragaloside II
S_AM_-8^C^	23.32			913.4772	−2.5	C_45_H_72_O_16_	Astragaloside I
S_AM_-9^C^	24.16			913.4762	−1.5	C_45_H_72_O_16_	Isoastragaloside I
S_AM_-10^C^	25.29			913.4772	−2.5	C_45_H_72_O_16_	Cyclosieversioside B
S_PG_-1^m^	2.79	979.5487	−3.5	1025.5474	3.5	C_44_H_84_O_23_	Q-*O*-Glc-Glc-Glc
S_PG_-2^m^	3.61	961.5381	−4.5			C_48_H_82_O_19_	Re1/Re2/Re3/20-glu-Rf/NotoginsenosideM/N/Vinaginsenoside R4
S_PG_-3^m^	3.65	961.5349	−2.4	1007.5427	0	C_48_H_82_O_19_	Re1/Re2/Re3/20-glu-Rf/NotoginsenosideM/N/Vinaginsenoside R4
S_PG_-4^m^	3.86	963.5538	0.9			C_48_H_84_O_19_	Neoalsoside J1
S_PG_-5^m^	5.22	961.5379	0.7			C_48_H_82_O_19_	Gypenoside Gc7
S_PG_-6^m^	7.02	961.5349	−2.4	847.5024	−3.7	C_48_H_82_O_19_	Re1/Re2/Re3/20-glu-Rf/NotoginsenosideM/N/Vinaginsenoside R4
S_PG_-7^m^	7.27	801.5007	0.9	847.5024	−3.7	C_42_H_74_O_14_	Ginsenoside Rf2
S_PG_-8^C^	7.71	931.5271	0.5	977.5328	0.7	C_47_H_80_O_18_	Re4/Notoginsenoside R1
S_PG_-9^C^	7.74	961.5372	0			C_48_H_82_O_19_	Re1/Re2/Re3/20-glu-Rf/NotoginsenosideM/N/Vinaginsenoside R4
S_PG_-10^C^	8.39	931.5237	−0.4	977.5336	1.2	C_47_H_80_O_18_	Re4/Notoginsenoside R1
S_PG_-11^m^	8.56	801.4427	0.2	847.4692	0.1	C_41_H_70_O_15_	Floralginsenoside C
S_PG_-12^m^	8.9	961.5391	−1.3			C_48_H_82_O_19_	Re1/Re2/Re3/20-glu-Rf/NotoginsenosideM/N/Vinaginsenoside R4
S_PG_-13^m^	9.01	961.5372	−2			C_48_H_82_O_19_	Re1/Re2/Re3/20-glu-Rf/NotoginsenosideM/N/Vinaginsenoside R4
S_PG_-14*^C^	9.2	799.4818	−3.3	845.4896	−0.4	C_42_H_72_O_14_	Ginsenoside Rg1
S_PG_-15*^C^	9.37	945.5444	2.2	991.5493	1.5	C_48_H_82_O_18_	Ginsenoside Re
S_PG_-16^C^	9.4	799.4818	−3.3	845.4896	−0.4	C_42_H_72_O_14_	Ginsenoside Rg1
S_PG_-17^m^	9.8	799.4863	2.4	845.4896	−0.4	C_42_H_72_O_14_	Isomer of ginsenoside Rg1
S_PG_-18^m^	10.12	799.4863	2.4	845.4896	−0.4	C_42_H_72_O_14_	Isomer of ginsenoside Rg1
S_PG_-19^m^	10.86	979.546	−1.8	1025.5508	−2.3	C_48_H_84_O_20_	Vinaginsenoside R13
S_PG_-20^m^	10.9	987.5534	0			C_50_H_84_O_19_	Acetyl-ginsenoside Re
S_PG_-21^m^	11.5	915.533	1.4	961.537	−0.2	C_47_H_80_O_17_	J/K/L-*O-*Xyl(Ara)-Rha-Glc
S_PG_-22^m^	11.63	987.5534	0			C_50_H_84_O_19_	Acetyl-ginsenoside Re
S_PG_-23^m^	12.56	915.5313	0.3	961.5377	0.5	C_47_H_80_O_17_	J/K/L-*O-*Glc-Rha-Xyl(Ara)
S_PG_-24^m^	12.72	1125.6024	−2.9	1171.6058	−4.6	C_54_H_94_O_24_	P-*O-*Glc-Glc-Glc-Glc
S_PG_-25^m^	13.09	987.5531	0.2	1033.5552	−3	C_50_H_84_O_19_	Acetyl-ginsenoside Re
S_PG_-26^m^	13.56	961.533	−4.4			C_48_H_82_O_19_	Re1/2/3, 20-glu-Rf, Notoginsenoside N
S_PG_-27^C^	14	785.4659	−3.6			C_41_H_70_O_14_	M/N/O-*O-*Glc-Xyl(Ara)
S_PG_-28^m^	14.24	963.5531	0.2			C_48_H_84_O_19_	Neoalsoside J1
S_PG_-29*^C^	14.46	799.4821	−2.9	845.4893	−0.7	C_42_H_72_O_14_	Ginsenoside Rf
S_PG_-30*^C^	14.57	799.4824	−2.5	845.4893	−0.7	C_42_H_72_O_14_	Pseudoginsenoside F11
S_PG_-31*^C^	15.24	769.4734	−0.5	815.4792	−0.1	C_41_H_70_O_13_	Notoginsenoside R2
S_PG_-32^m^	15.99	637.4327	1.7	683.4374	0.6	C_36_H_62_O_9_	Ginsenoside Rh1
S_PG_-33*^C^	16.08	783.4918	2.3	829.4974	3	C_42_H_72_O_13_	Ginsenoside Rg2
S_PG_-34^C^	16.38	783.4894	−0.1	829.4948	−0.1	C_42_H_72_O_13_	Isomer of ginsenoside Rg2
S_PG_-35*^C^	16.5	637.4327	1.7	683.4374	0.6	C_36_H_62_O_9_	Ginsenoside Rh1
S_PG_-36^m^	16.77	1029.6227	−3.4	1255.6305	−1.4	C_58_H_98_O_26_	G/H-*O-*Glc-Glc-Glc-Xyl(Ara)-Xyl(Ara)
S_PG_-37*^C^	16.97	1107.5946	−0.5	1153.6022	1.4	C_54_H_92_O_23_	Ginsenoside Rb1
S_PG_-38^m^	17.32	1149.6062	0.4	1195.6072	−3.3	C_56_H_94_O_24_	Quinquenoside R1
S_PG_-39*^C^	17.51	955.4901	−0.2			C_48_H_76_O_19_	Ginsenoside Ro
S_PG_-40*^C^	17.51	1077.5848	0.3			C_53_H_90_O_22_	Ginsenoside Rc
S_PG_-41^C^	17.63	1209.6207	−5			C_58_H_98_O_26_	Ginsenoside Ra1
S_PG_-42^C^	17.88	1119.5918	−2.9	1165.5959	−4	C_55_H_92_O_23_	Ginsenoside Rs2
S_PG_-43*^C^	18.09	1077.5822	3.2	1123.5898	−0.2	C_53_H_90_O_22_	Ginsenoside Rb2
S_PG_-44*^C^	18.28	1077.5822	−2.5			C_53_H_90_O_22_	Ginsenoside Rb3
S_PG_-45^C^	18.37	1119.5936	−1.3	1165.5957	−4.2	C_55_H_92_O_23_	Ginsenoside Rs2
S_PG_-46^C^	18.74	1149.6062	0.4	1195.6072	−3.3	C_56_H_94_O_24_	Quinquenoside R1
S_PG_-47^m^	19.1	793.439	2			C_42_H_66_O_14_	Chikusetsusaponin Iva/Zingibroside R1
S_PG_-48*^C^	19.23	945.5413	−1.1	991.5466	−1.2	C_48_H_82_O_18_	Ginsenoside Rd
S_PG_-49^C^	19.25	1119.5918	−2.9	1165.5959	−4	C_55_H_92_O_23_	Ginsenoside Rs2
S_PG_-50^m^	19.53	987.5536	0.7	1033.5531	−5	C_50_H_84_O_19_	Pseudoginsenoside Rc1
S_PG_-51^m^	19.54	1031.5437	1			C_51_H_84_O_21_	Malonyl-Ginsenoside Rd
S_PG_-52^C^	19.54	945.5413	−1.1	991.5466	−1.2	C_48_H_82_O_18_	Isomer of ginsenoside Rd
S_PG_-53^m^	19.73	987.5536	0.7	1033.5531	−5	C_50_H_84_O_19_	Pseudoginsenoside Rc1
S_PG_-54^C^	19.77	945.5413	−1.1	991.5466	−1.2	C_48_H_82_O_18_	Isomer of ginsenoside Rd
S_PG_-55^C^	19.79	1119.5936	−1.3	1165.5957	−4.2	C_55_H_92_O_23_	Ginsenoside Rs2
S_PG_-56^C^	20.17	945.5433	1.1	991.5486	0.8	C_48_H_82_O_18_	Gypenoside XVII
S_PG_-57^C^	20.35	987.5536	0.7	1033.5531	−5	C_50_H_84_O_19_	Pseudoginsenoside Rc1
S_PG_-58^m^	20.75	915.5294	−0.3	961.5387	1.6	C_47_H_80_O_17_	G/H-*O-*Glc-Glc-Xyl(Ara)
S_PG_-59^C^	21.19	987.5536	0.7	1033.5531	−5	C_50_H_84_O_19_	Pseudoginsenoside Rc1
S_PG_-60^m^	21.25	751.4615	−2.4			C_41_H_68_O_12_	Notoginsenoside T5
S_PG_-61^m^	21.75	751.4645	1.6			C_41_H_68_O_12_	Notoginsenoside T5
S_PG_-62*^C^	21.79	765.4765	−3.1	811.4833	−1.4	C_42_H_70_O_12_	Ginsenoside Rg6
S_PG_-63*^C^	22.27	765.4797	1	811.4847	0.4	C_42_H_70_O_12_	Ginsenoside F4
S_PG_-64^C^	23.04	783.4885	−1.3	829.4949	0	C_42_H_72_O_13_	Isomer of ginsenoside Rg3
S_PG_-65^m^	23.5	793.4382	1			C_42_H_66_O_14_	Chikusetsusaponin Iva/Zingibroside R1
S_PG_-66*^C^	24.4	783.4885	−1.3	829.4949	0	C_42_H_72_O_13_	Ginsenoside Rg3
S_PG_-67^C^	24.72	783.4885	−1.3	829.4949	0	C_42_H_72_O_13_	Isomer of ginsenoside Rg3
S_PG_-68*^C^	27.55	765.4778	−1.4	811.4836	−1	C_42_H_70_O_12_	Ginsenoside Rk1
S_PG_-69*^C^	27.88	765.4782	−0.9	811.4824	−2.5	C_42_H_70_O_12_	Ginsenoside Rg5
S_PG_-70^m^	28	807.485	−4.5			C_44_H_72_O_13_	Ginsenoside Rs4/Rs5
S_PG_-71^m^	28.2	621.4378	1.9	667.4397	−3.6	C_36_H_62_O_8_	Ginsenoside Rh2
S_PG_-72 *^m^	28.41	621.4396	4.8	667.4421	0	C_36_H_62_O_8_	Ginsenoside Rh2
S_PG_-73^m^	29.57	807.4892	−0.4	853.4925	−2.8	C_44_H_72_O_13_	Ginsenoside Rs4/Rs5
F_AM_-8^m^	6.4	595.1461	1.5			C_27_H_32_O_15_	5/8-Hydroxy-liquiritigenin-*O*-diglucoside or 8-Hydroxy-liquiritigenin-*O*-diglucoside
F_AM_-13^C^	7.46	461.1081	−0.7			C_22_H_22_O_11_	Isomer of 5′-hydroxy-4′-methoxyisoflavone-3′-*β*-d-glucoside
F_AM_-36*^m^	10	595.2013	−2.4			C_28_H_36_O_14_	Isomucronulatol-2′-*O*-*β*-d-apiosyl(1 → 2)-*β*-d-glucoside
F_AM_-38^m^	10.82	431.0975	−0.7			C_21_H_20_O_10_	5,7-Dihydroxyl-flavone-4′-*O*-glucoside
F_AM_-40^m^	11.21	461.1069	−3.3			C_22_H_22_O_11_	Isomer of 5′-hydroxy-4′-methoxyisoflavone-3′-*β*-d-glucoside
F_AM_-41^m^	11.25	445.1124	−2.5	491.1194	0.8	C_22_H_22_O_10_	Isomer of calycosin-7-*O*-*β*-d-glucoside
F_AM_-43^m^	11.44	625.2121	−1.8			C_29_H_38_O_15_	Isomucronulatol-*O*-diglucoside
F_AM_-47^m^	11.6	447.1281	−2.2			C_22_H_24_O_10_	5-Hydroxy-7-methoxyflavanone-5-*O*-glucoside
F_AM_-49^m^	12.1	463.1617	2.8	509.1708	−0.1	C_23_H_28_O_10_	Isomucronulatol-7-*O*-*β*-d-glucoside
F_AM_-50^m^	12.28	433.1477	−3.4			C_22_H_26_O_9_	Isomucronulatol-*O*-apioside
F_AM_-54^m^	12.8	253.0498	−0.12			C_15_H_10_O_4_	Isomer of 7,4′-dihydroxyflavone
F_AM_-60^m^	13.3	463.1617	2.8	509.1708	−0.1	C_23_H_28_O_10_	Isomucronulatol-7-*O*-*β*-d-glucoside
F_AM_-62^m^	13.6	283.0607	−0.1			C_16_H_12_O_5_	Isomer of calycosin
F_AM_-64*^m^	13.7	253.0498	−0.12			C_15_H_10_O_4_	7,4′-Dihydroxyflavone
F_AM_-67^m^	13.91	471.1291	−0.6	517.134	−1.1	C_24_H_24_O_10_	Acetyl-ononin
F_AM_-68^m^	14.2	283.0616	−3.6			C_16_H_12_O_5_	Isomer of calycosin
F_AM_-69^m^	14.22	579.1721	1.2			C_27_H_32_O_14_	Liquiritigenin/Isoliquiritigenin-*O*-diglucoside
F_AM_-70^m^	14.23	593.1862	−1.3			C_28_H_34_O_14_	9,10-Dimethoxy-pterocarpane-3-*O*-glucoside-apioside
F_AM_-71^m^	14.47	593.1862	−1.3			C_28_H_34_O_14_	3,9-Dimethoxy-pterocarpane-10-*O*-glucoside-apioside
F_AM_-72^C^	15.07	463.1617	2.8	509.1708	−0.1	C_23_H_28_O_10_	Isomucronulatol-7-*O*-*β*-d-glucoside
F_AM_-75*^C^	15.22	283.0607	−0.1			C_16_H_12_O_5_	Calycosin
F_AM_-76*^C^	15.7	463.1617	2.8	509.1708	−0.1	C_23_H_28_O_10_	(3 *R*)-(+)-isomucronulatol-2′-*O*-*β*-d-glucoside
F_AM_-77^m^	17.87	269.0451	0.4			C_15_H_10_O_5_	Resokaempferol
F_AM_-78^m^	18.3	299.0562	1			C_16_H_12_O_6_	Isomer of pratensein
F_AM_-79^m^	18.9	299.0899				C_17_H_16_O_5_	3-Hydroxy-9,10-dimethoxy-pterocarpane
F_AM_-80^m^	19.01	299.0899	1			C_17_H_16_O_5_	10-Hydroxy-3,9-dimethoxy-pterocarpane
F_AM_-81^C^	19.09	253.0498	−0.12	299.0567	3.7	C_15_H_10_O_4_	Isomer of 7,4′-dihydroxyisoflavone
F_AM_-82*^C^	20.25	267.0658	0.4			C_16_H_12_O_4_	Formononetin
F_CC_-3^C^	4.97	289.0727	4.1			C_15_H_14_O_6_	Catechin or Epicatechin
F_CC_-9^C^	6.65	289.072	3.1			C_15_H_14_O_6_	Catechin or Epicatechin
F_CC_-11^m^	6.69	289.0727	4.1			C_15_H_14_O_6_	Catechin or Epicatechin
F_GU,AM_-25*^C^	8.95	445.1125	−2.3	491.1192	0	C_22_H_22_O_10_	Calycosin-7-*O*-*β*-d-glucoside
F_GU,AM_-51^m^	12.3	561.1617	1.6			C_27_H_30_O_13_	Isomer of ononin-*O*-apioside
F_GU,AM_-55^m^	13	561.1617	1.6			C_27_H_30_O_13_	Isomer of ononin-*O*-apioside
F_GU_-2^m^	3.95	661.178	2.3			C_32_H_34_O_16_	Isomer of liquiritigenin-*O*-diglucoside
F_GU_-4^m^	5.7	499.1238	−1.2			C_25_H_24_O_11_	Isomer of liquiritin
F_GU_-5^m^	5.87	499.1248	0.9			C_25_H_24_O_11_	Isomer of liquiritin apioside
F_GU_-6^m^	5.88	631.167	1			C_30_H_32_O_15_	Isomer of liquiritin apioside
F_GU_-7^C^	6.18	579.1732	3.1	625.1782	2.1	C_27_H_32_O_14_	Isomer of liquiritigenin-*O*-diglucoside
F_GU_-10^m^	6.65	711.2163	2.7			C_32_H_40_O_18_	Isomer of liquiritin-*O*-glucoside
F_GU_-12*^C^	7.21	593.1513	1.2			C_27_H_30_O_15_	Apigenin-6,8-di-*C*-*β*-d-glucopyranoside
F_GU_-14^m^	7.66	415.1016	−2.9			C_21_H_20_O_9_	Daidzein-7-O-galactoside
F_GU_-15^m^	7.7	595.166	−0.5	641.1732	2.2	C_27_H_32_O_15_	5-Hydroxy-liquiritigenin-*O*-diglucoside or 8-Hydroxy-liquiritigenin-*O*-diglucoside
F_GU_-16^C^	8.1	563.1408	1.2			C_26_H_28_O_14_	Isoschaftoside
F_GU_-17^C^	8.2	579.1732	3.1			C_27_H_32_O_14_	Liquiritigenin-*O*-diglucoside
F_GU_-18^m^	8.34	711.2163	2.7			C_32_H_40_O_18_	5-Dihydroxy-liquiritigenin-*O*-diglucoside or 8-Dihydroxy-liquiritigenin-*O*-diglucoside
F_GU_-19^m^	8.41	415.1016	−2.9			C_21_H_20_O_9_	Daidzein-7-*O*-galactoside
F_GU_-20^m^	8.42	547.1432	−2.4			C_26_H_28_O_13_	Liquiritin/Isoliquiritin-*O*-apioside
F_GU_-21^m^	8.64	711.2163	2.7			C_32_H_40_O_18_	5-Dihydroxy-liquiritigenin-*O*-diglucoside or 8-Dihydroxy-liquiritigenin-*O*-diglucoside
F_GU_-22^m^	8.75	547.1432	−2.4			C_26_H_28_O_13_	Liquiritin/Isoliquiritin-*O*-apioside
F_GU_-23^C^	8.75	433.1129	−1.4			C_21_H_22_O_10_	5-Hydroxy-liquiritin or 8-Hydroxy-liquiritin
F_GU_-24^C^	8.82	579.1732	3.1			C_27_H_32_O_14_	Liquiritigenin-*O*-diglucoside
F_GU_-26*^C^	8.96	417.1188	0.5			C_21_H_22_O_9_	Neoisoliquiritin
F_GU_-27^C^	9	579.1732	3.1			C_27_H_32_O_14_	Liquiritigenin-*O*-diglucoside
F_GU_-28^m^	9.01	433.1135	0			C_21_H_22_O_10_	5-Hydroxy-liquiritin or 8-Hydroxy-liquiritin
F_GU_-29^C^	9.02	549.1609	0.2			C_26_H_30_O_13_	Neoliquiritin-*O*-apioside
F_GU_-30*^C^	9.11	417.1187	0.2			C_21_H_22_O_9_	Liquiritin
F_GU_-31*^C^	9.4	549.1609	0.2			C_26_H_30_O_13_	Liquiritin apioside
F_GU_-32^m^	9.51	447.1308	3.1			C_22_H_24_O_10_	2′-Hydroxy-7-methoxyflavanone-5-*O*-glucoside
F_GU_-33^C^	9.61	577.1555	0.7			C_27_H_30_O_14_	Isoviolanthin/Violanthin
F_GU_-34^m^	9.7	565.1555	−0.4			C_26_H_30_O_14_	7,8-Dihydroxyl-flavanone-4′-*O*-*β*-d-apiofuranosyl(1′ → 2″)-*O*-glucoside
F_GU_-35^m^	9.7	447.1304	2.9			C_22_H_24_O_10_	5-Hydroxy-7-methoxyflavanone-5-*O*-glucoside
F_GU_-37^m^	10.6	417.1187	0.2			C_21_H_22_O_9_	Isomer of liquiritin
F_GU_-39^m^	11	447.1294	1.2			C_22_H_24_O_10_	Genistin
F_GU_-42^m^	11.25	447.1307	3			C_22_H_24_O_10_	5-Hydroxy-7-methoxyflavanone-5-*O*-glucoside
F_GU_-44^m^	11.48	565.1575	3.2			C_26_H_30_O_14_	7,8-Dihydroxyl-flavanone-4′-*O*-*β*-d-apiofuranosyl(1′ → 2″)-*O*-glucoside
F_GU_-45^C^	11.48	433.1127	−1.8			C_21_H_22_O_10_	5-Hydroxy-liquiritin or 8-Hydroxy-liquiritin
F_GU_-46^m^	11.5	579.1711	−0.3			C_27_H_32_O_14_	Isomer of liquiritigenin-*O*-diglucoside
F_GU_-48^m^	11.9	549.1609	0.2			C_26_H_30_O_13_	Liquiritin apioside
F_GU_-52^m^	12.4	431.1345	−0.7			C_22_H_23_O_9_	Genistin
F_GU_-53^m^	12.75	445.1124	−2.5	491.1194	0.8	C_22_H_22_O_10_	Isomer of calycosin-7-*O*-*β*-d-glucoside
F_GU_-56^m^	13	579.1721	1.2			C_27_H_32_O_14_	Isomer of isoliquiritigenin-*O*-diglucoside
F_GU_-57*^C^	13.1			475.1263	2.3	C_22_H_22_O_9_	Ononin
F_GU_-58^m^	13.1	591.1698	−2.7			C_28_H_32_O_14_	Acetyl-isoliquiritin-*O*-apioside
F_GU_-59*^C^	13.21	549.1609	0.2			C_26_H_30_O_13_	Isoliquiritin apioside
F_GU_-61*^C^	13.5	417.1187	0.2			C_21_H_22_O_9_	Isoliquiritin
F_GU_-63^m^	13.63	459.1301	2			C_23_H_24_O_10_	Acetyl-liquiritin/isoliquiritin
F_GU_-65*^C^	13.81	549.1609	0.2			C_26_H_30_O_13_	Licuraside
F_GU_-66*^C^	13.9	255.0656	−0.4			C_15_H_12_O_4_	Liquiritigenin
F_GU_-73^m^	15.13	433.151	2.5			C_22_H_26_O_9_	5-Hydroxy-liquiritin or 8-Hydroxy-liquiritin
F_GU_-74^m^	15.20	695.1835	−1.3			C_31_H_36_O_18_	Liquiritigenin/Isoliquiritigenin-*O*-Glc-Api-Rha
F_GU_-79^m^	18.56	459.13	2			C_23_H_24_O_10_	Acetyl-Liquiritin/Isoliquiritin
F_GU_-83*^C^	20.26	255.0656	−0.4			C_15_H_12_O_4_	Isoliquiritigenin
P_CC_-1^C^	2.98	577.1349	−0.3			C_30_H_26_O_16_	PAC B-type dimer
P_CC_-2^C^	3.73	577.1353	0.2			C_30_H_26_O_16_	PAC B-type dimer
P_CC_-3^C^	4.14	577.1343	−1.4			C_30_H_26_O_16_	PAC B-type dimer
P_CC_-4^C^	4.54	577.1347	-0.7			C_30_H_26_O_16_	PAC B-type dimer
P_CC_-5^C^	5.07	577.1345	−0.9			C_30_H_26_O_16_	PAC B-type dimer
P_CC_-6^C^	6.46	865.2001	1.5			C_45_H_38_O_18_	PAC B-type trimer
L_CC_-1^C^	6.02	653.2457	0.9			C_31_H_42_O_15_	Isolariciresinol-4-*O*-*β*-d-apiosyl (1 → 2)-*β*-d-glucoside
L_CC_-2^C^	6.05	653.2453	1.3			C_31_H_42_O_15_	Isolariciresinol-3′-*O*-*β*-d-apiosyl (1 → 2)-*β*-d-glucoside
L_CC_-3^C^	6.29	551.2119	−2.7			C_27_H_36_O_12_	5-Methoxy-isolariciresinol-4-*O*-*β*-d-glucoside
L_CC_-4^C^	6.39	521.2019	−0.8			C_26_H_34_O_11_	Isolariciresinol-4-*O*-*β*-d-glucoside
D_CC_-1^C^	3.04	543.2439	−0.2			C_26_H_40_O_12_	Cinncaside
D_CC_-2*^C^	3.85	365.1955	−0.9			C_20_H_30_O_6_	Anhydrocinnzeylanol
D_CC_-3*^C^	4.51	425.2178	0.3			C_22_H_34_O_8_	Cinnzeylanine
D_CC_-4*^C^	9.10	407.2014	−0.7			C_22_H_32_O_7_	Anhydrocinnzeylanine

Notes *: the compound identified by comparison with the reference.

^a^: the single English alphabet in capital or in lowercase means the aglycone in Fig. S5.

^C^: the compound identified by UNIFI.

^m^: the compound detected by multiple screening modes of Qtrap-MS.

F: flavonoid; S: saponin; P: procyanidin; L: lignan; D: diterpene.

_AM_: the compound originated from *A. membranceus*; _GU_: the compound originated from *G. uralensis*; _PG_: the compound originated from *P. ginseng*; and _CC_: the compound originated from *C. cassia*.

**Table 2:** The contents (mg/g) of 36 investigated compounds in six batches of BYD extracts.

No.	Analytes	Batch 1 (mg/g)	Batch 2 (mg/g)	Batch 3 (mg/g)	Batch 4 (mg/g)	Batch 5 (mg/g)	Batch 6 (mg/g)
1	Apigenin-6,8-di-*C*-*β*-d-glucopyranoside	38.79	35.78	37.85	38.79	39.85	35.67
2	Calycosin-7-*O*-*β*-d-glucoside	93.47	89.45	95.62	91.48	93.33	95.68
3	Liquiritin	160.86	159.86	168.86	165.86	161.86	169.86
4	Liquiritin aposide	567.75	534.65	558.68	561.83	568.70	578.01
5	Isoliquiritin aposide	147.46	153.43	133.71	149.48	162.72	156.80
6	Ginsenoside Rg1	521.08	500.76	489.19	499.81	473.48	514.48
7	Ginsenoside Re	542.16	557.93	572.29	583.85	572.33	589.62
8	Isoliquiritin	249.15	265.10	235.58	269.19	258.59	261.74
9	Liquiritigenin	74.41	68.23	64.53	80.42	76.53	74.57
10	Ononin	104.94	114.92	125.13	118.95	107.13	115.19
11	Calycosin	129.62	138.59	131.84	125.63	133.85	141.2
12	Uralsaponin C	167.29	153.51	149.32	169.2	163.75	159.42
13	Uralsaponin F	131.85	132.03	128.88	139.78	121.43	127.96
14	Licorice saponin A3	31.85	29.03	30.88	32.78	31.43	32.96
15	Ginsenoside Rf	98.64	90.77	104.66	101.59	93.32	97.72
16	22*β*-Acetoxyglycyrrhizin	279.58	283.97	285.65	269.43	281.67	273.81
17	Ginsenoside Rg2	554.16	572.93	549.29	569.85	567.33	583.62
18	Ginsenoside Rh1	15.80	13.50	16.10	154.00	14.80	14.10
19	Ginsenoside Rb1	785.87	771.93	769.05	791.45	778.37	786.50
20	Licorice saponin E2	159.97	151.19	149.01	161.89	163.47	153.10
21	Licorice saponin H2	9.67	8.99	9.32	9.69	9.78	10.01
22	Ginsenoside Rc	349.33	352.81	357.41	361.14	347.20	359.62
23	Licorice saponin G2	83.78	88.90	84.80	87.73	86.50	82.85
24	Isoliquiritigenin	6.14	6.63	6.54	5.92	5.76	6.06
25	Ginsenoside Ro	710.57	718.55	697.73	717.18	725.28	712.16
26	Ginsenoside Rb2	210.32	202.61	193.37	219.21	222.64	213.50
27	Formononetin	51.79	54.78	49.88	52.83	58.88	53.91
28	Ginsenoside Rb3	42.14	40.82	42.95	41.18	42.93	39.85
29	Ginsenoside Rg3	279.16	572.93	549.29	569.85	567.33	583.62
30	Ginsenoside Rd	90.21	85.34	99.23	101.15	84.90	95.28
31	Astragaloside II	194.92	205.20	214.97	197.81	221.27	215.09
32	Isoastragaloside II	87.33	90.95	86.54	91.37	88.34	86.39
33	Glycyrrhizinic acid	529.06	517.78	533.18	549.77	558.36	530.49
34	Ginsenoside F4	25.23	24.71	23.94	26.25	24.99	27.14
35	Astragaloside IV	702.02	691.91	689.80	711.72	703.45	709.86
36	Ginsenoside Rg5	46.23	42.79	47.19	49.12	43.27	46.29
