# Supplementary material for: An Integrated Strategy for Global Qualitative and Quantitative Profiling of Traditional Chinese Medicine Formulas: Baoyuan Decoction as a Case
Source: Sci Rep. 2016 Dec 7;6:38379. doi: 10.1038/srep38379 (PMC5141425; doi:10.1038/srep38379)
Supplement: Supplementary Information [file srep38379-s1.pdf]

Supplementary data

An Integrated Strategy for Global Qualitative and Quantitative Profiling of Traditional Chinese Medicine Formulas: *Baoyuan* Decoction as a Case

Xiaoli Ma[1,*] Xiaoyu Guo[1,*], Yuelin Song[2], Lirui Qiao[3], WenguangWang[1], Mingbo Zhao[1], Pengfei Tu[1] & Yong Jiang[1]

[1]State Key Laboratory of Natural and Biomimetic Drugs, School of Pharmaceutical Sciences, Peking University, Beijing 100191, People's Republic of China.

[2]Modern Research Center for Traditional Chinese Medicine, Beijing University of Chinese Medicine, Beijing 100029, People's Republic of China.

[3]Waters corporation Shanghai Science & Technology Co Ltd, Shanghai 201206, People's Republic of China.

[*]These two authors contributed equally.

Correspondence and requests for materials should be addressed to Y. J. (yongjiang@bjmu.edu.cn).

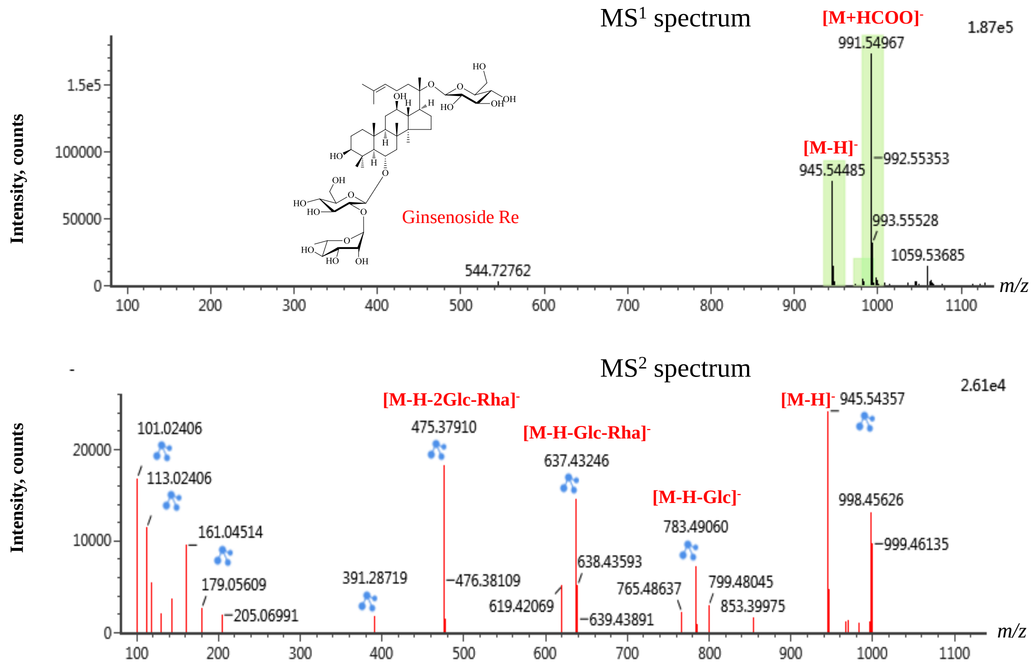

Figure S1 The accurate MS and MS/MS spectra and the chemical structure of ginsenoside Re. The signals either highlighted in green or tagged in blue were chosen by UNIFI for rapid structural identification.

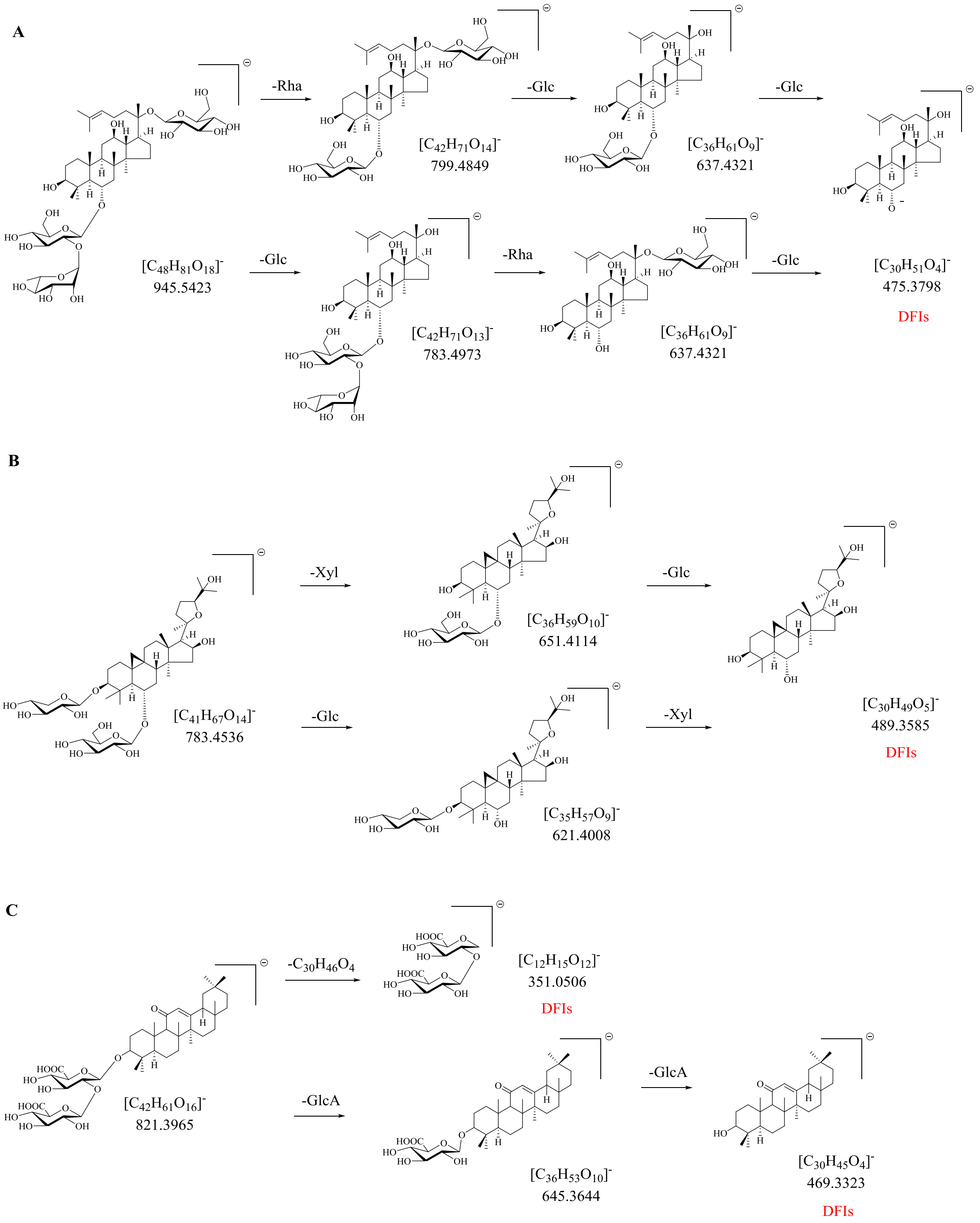

Figure S2 The proposed fragmentation pathways and main diagnostic fragment ions (DFIs) for ginsenoside Re (A), astragaloside IV (B), and glycyrrhizic acid (C).

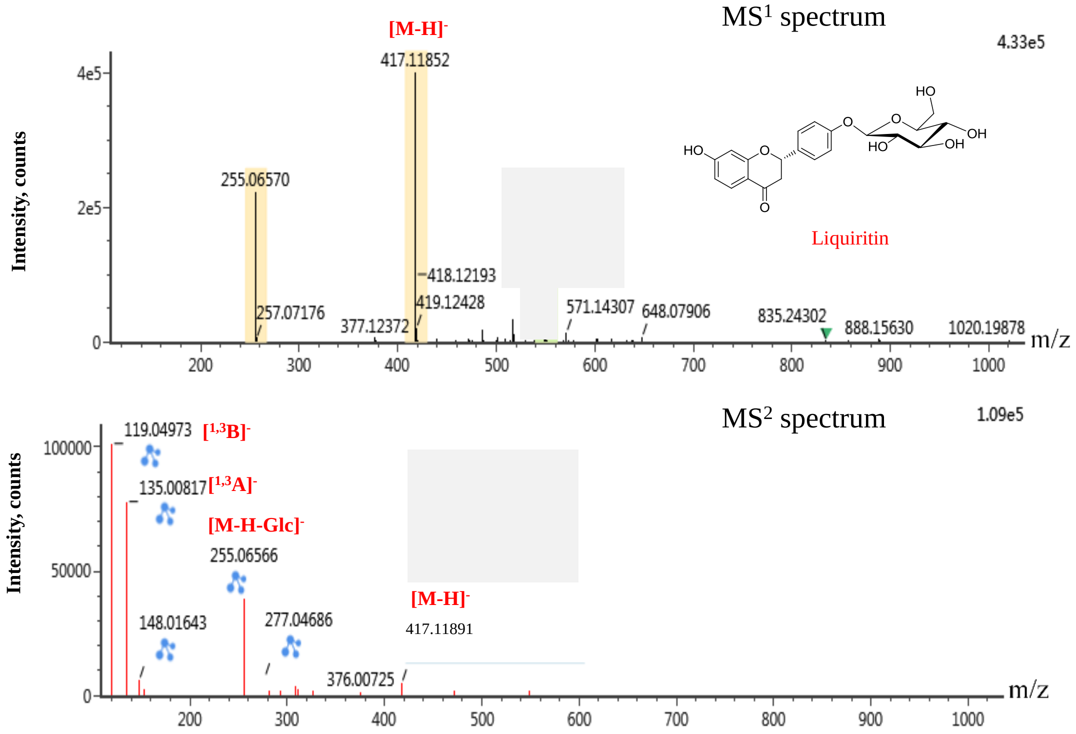

Figure S3 The accurate MS and MS/MS spectra and the chemical structure of liquiritin. The signals either highlighted in yellow or tagged in blue were chosen by UNIFI for rapid structural identification.

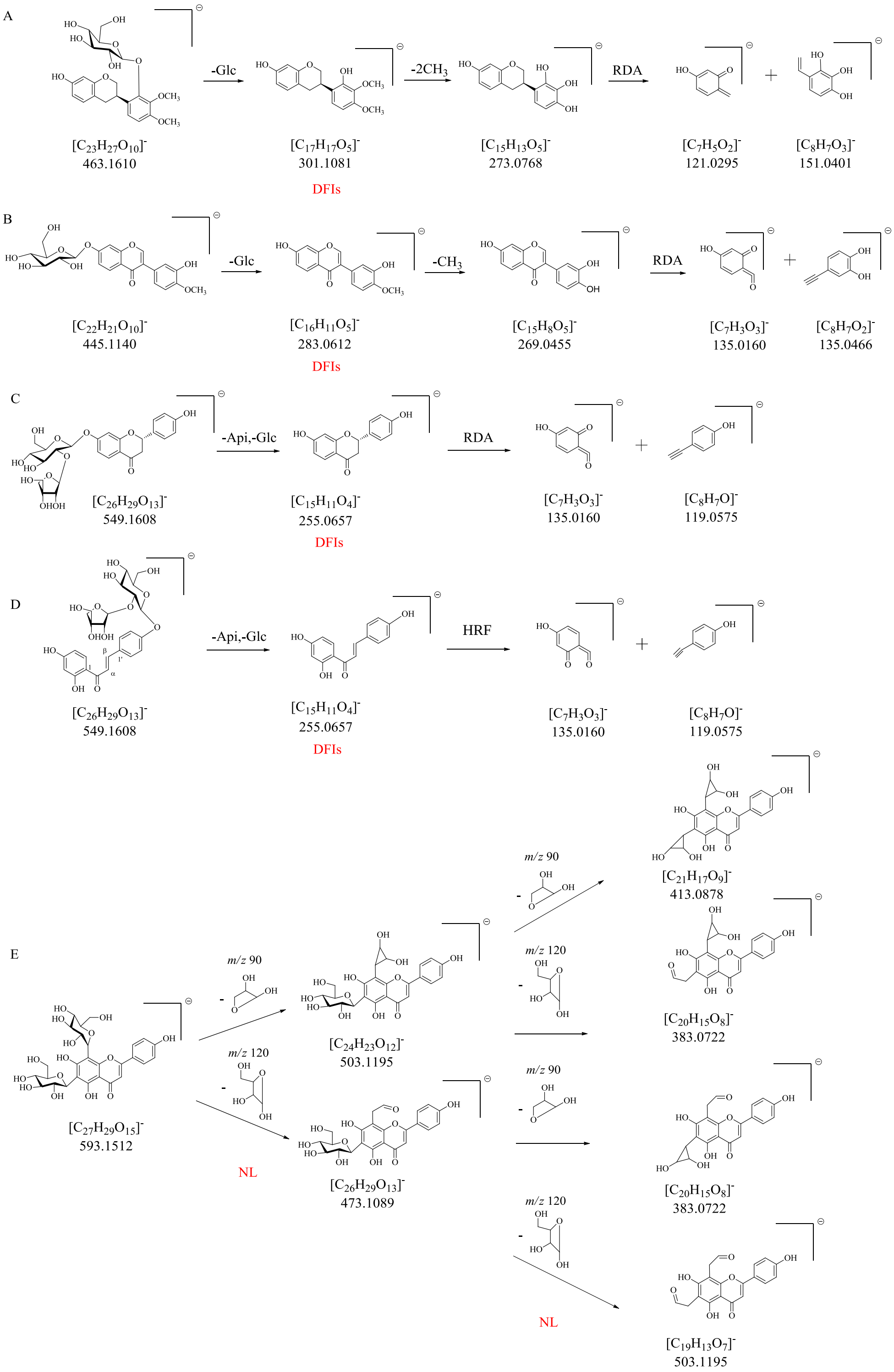

Figure S4 The proposed fragmentation pathways and primary diagnostic fragment ions (DFIs) for liquiritin apioside (A), isoliquiritin apioside (B), calycosin-7-*O*-*β*-D-glucoside (C), (3*R*)-(+)- isomucronulatol-2′-*O*-*β*-D-glucopyranoside (D), and apigenin-6,8-di-*C*-*β*-D-glucopyranoside (E).

The aglycones of ginsenosides:

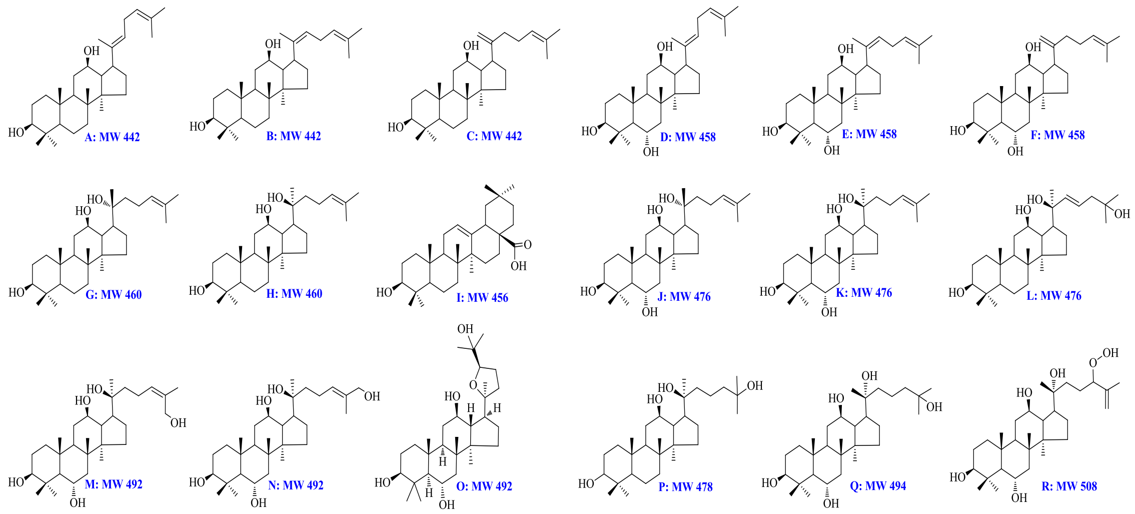

The aglycone of astragalosides:

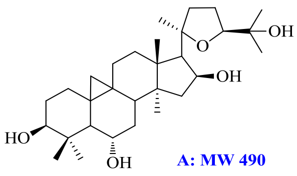

The aglycones of licorice saponins:

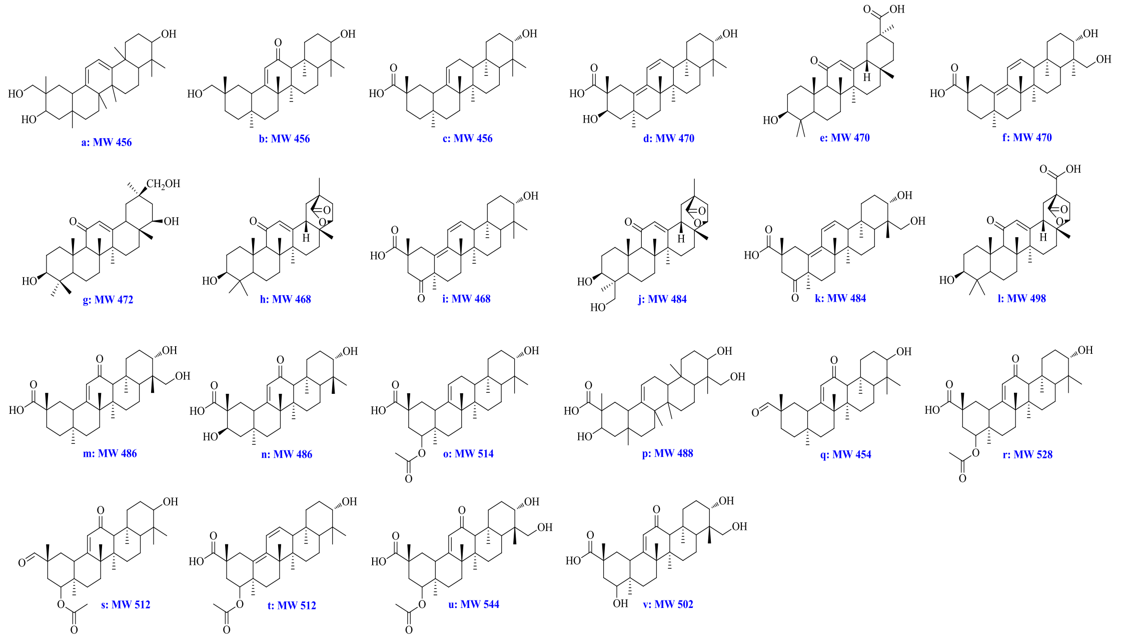

The aglycones of flavonoids:

Aglycone	*m/z*	Predictive formula	Characteristic fragments	Group	OH	OCH_3
FA-1	255.0663	$C_{15}H_{11}O_4$	135.01,119.05	I	2	0
FA-2	271.0612	$C_{15}H_{11}O_5$	151.00,119.05	I	3	0
FA-3	253.0506	$C_{15}H_9O_4$	135.02,117.04	II	2	0
FA-4	267.0663	$C_{16}H_{11}O_4$	252.04,193.05,135.01	II	1	1
FA-5	269.0455	$C_{15}H_9O_5$	151.00,117.03	II	3	0
FA-6	269.0814	$C_{16}H_{13}O_4$	270.05, 119.05	I	2	1
FA-7	283.0612	$C_{16}H_{11}O_5$	268.03,193.05,135.01	II	2	1
FA-8	285.0612	$C_{16}H_{11}O_5$	270.05,119.05	I	2	1
FA-9	289.0712	$C_{15}H_{13}O_6$	137.0238,109.0196	III	5	0
FA-10	299.0561	$C_{16}H_{11}O_6$	284.03,135.02	II	3	1
FA-11	299.0925	$C_{17}H_{15}O_5$	284.07,269.04	IV	1	2
FA-12	301.1099	$C_{17}H_{17}O_5$	271.06,193.05,135.05	III	3	2

Group I:

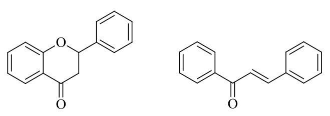

Group II:

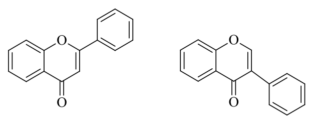

Group III:

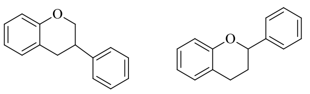

Group IV:

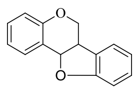

Figure S5 The common aglycones of ginsenosides, astragalosides, licorice saponins, and flavonoids in BYD.

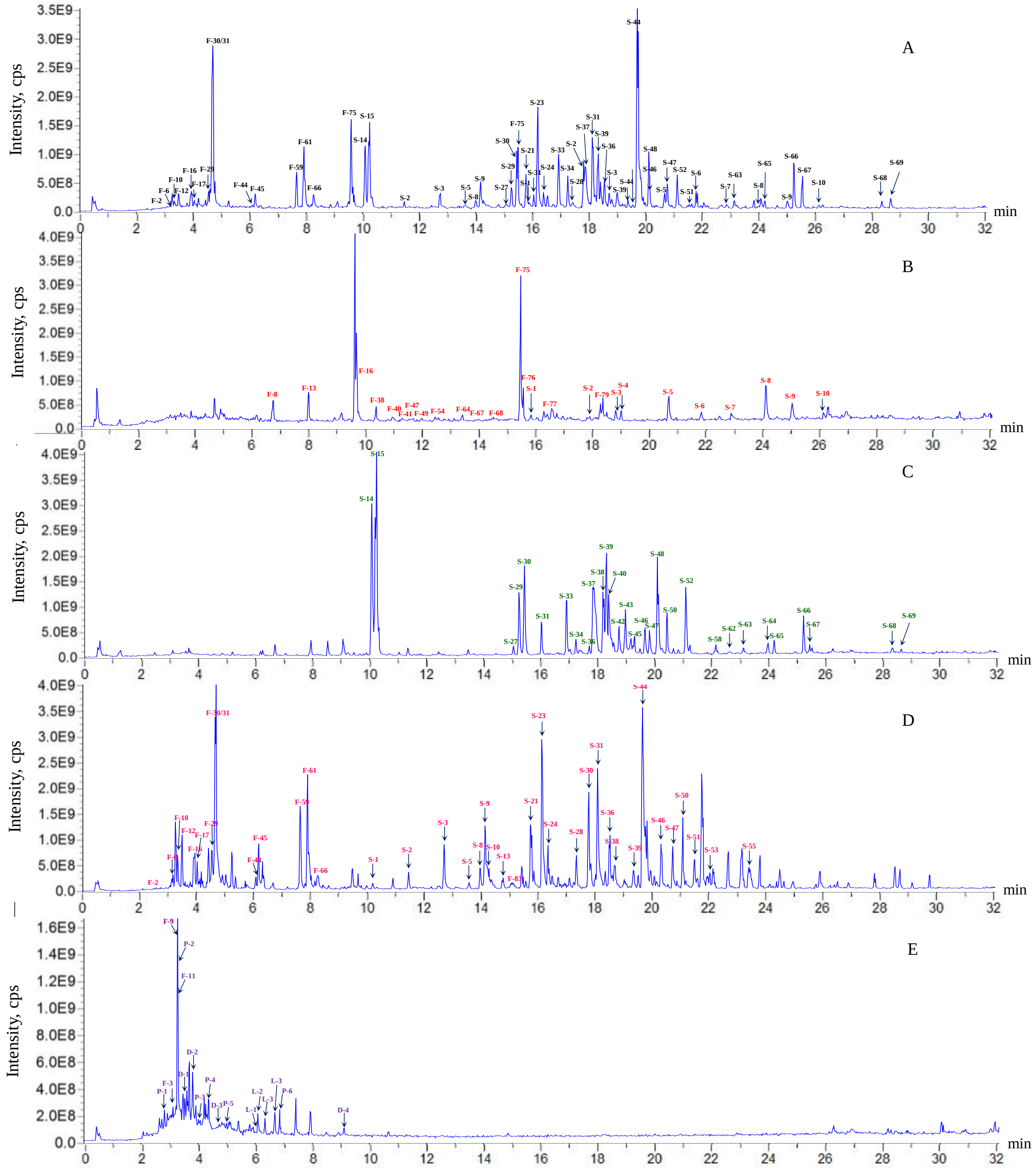

Figure S6 The base peak chromatograms (BPCs) of *Baoyuan* decoction extract (A), Astragali Radix extract (B), Ginseng Radix extract (C), Glycyrrhiza Radix et Rhizoma Praeparata Cum Melle extract (D), and Cinnamomi Cortex extract (E) by UPLC/Qtrap-MS in negative mode.

Table S1 The MS/MS spectral data of chemical constituents of *Baoyuan* decoction by UPLC/Q-TOF-MS.

No.	t_R (min)	$[Aglycone-H]^-$	Fragment ions
S_{GU}-1[m]	9.5	p $[M-H-2GlcA]^-$	663.3879 $[M-H-GlcA]^-$, 351.0556 $[2GlcA-H]^-$
S_{GU}-2[m]	10.94	g $[M-H-2GlcA-Rha]^-$	793.4360 $[M-H-GlcA]^-$, 617.3657 $[M-H-2GlcA]^-$, 471.3477 $[M-H-2GlcA-Rha]^-$, 351.0558 $[2GlcA-H]^-$
S_{GU}-3*[C]	12.2	g $[M-H-2GlcA]^-$	647.3791 $[M-H-GlcA]^-$, 471.3448 $[M-H-2GlcA]^-$, 351.0563 $[2GlcA-H]^-$
S_{GU}-4[m]	12.33	g $[M-H-2GlcA-Rha]^-$	823.4045 $[M-H-Rha]^-$, 471.3477 $[M-H-2GlcA-Rha]^-$, 351.0558 $[2GlcA-H]^-$
S_{GU}-5[m]	12.82	g $[M-H-2GlcA-Glc- C_2H_2O]^-$	985.4524 $[M-H-C_2H_2O]^-$, 823.3965 $[M-H-C_2H_2O-Glc]^-$, 471.3505 $[M-H-2GlcA-Glc-C_2H_2O]^-$, 351.0581 $[2GlcA-H]^-$
S_{GU}-6[C]	12.97	j/k $[M-H-2GlcA]^-$	659.3494 $[M-H-GlcA]^-$, 483.3309 $[M-H-2GlcA]^-$, 351.0563 $[2GlcA-H]^-$
S_{GU}-7 [m]	13.06	g $[M-H-2GlcA]^-$	647.3791 $[M-H-GlcA]^-$, 471.3448 $[M-H-2GlcA]^-$, 351.0563 $[2GlcA-H]^-$
S_{GU}-8 [m]	13.4	m/n $[M-H-2GlcA-Gic]^-$	837.3893 $[M-H-Glc]^-$, 661.3572 $[M-H-GlcA-Glc]^-$, 485.3264 $[M-H-2GlcA-Glc]^-$, 351.0562 $[2GlcA-H]^-$
S_{GU}-9*	13.5	u $[M-H-2GlcA]^-$	719.3635 $[M-H-GlcA]^-$, 351.0554 $[2GlcA-H]^-$
S_{GU}-10[C]	13.6	d/e/f $[M-H-2GlcA]^-$	645.3670 $[M-H-GlcA]^-$, 469.3323 $[M-H-2GlcA]^-$, 425.3443 $[M-H-2GlcA-CO_2]^-$, 351.0577 $[2GlcA-H]^-$
S_{GU}-11[C]	13.68	v $[M-H-2GlcA]^-$	501.3202 $[M-H-2GlcA]^-$
S_{GU}-12 [m]	14.07	499.3 $[M-H-2GlcA]^-$	851.4082 $[M-H-GlcA]^-$, 499.3202 $[M-H-2GlcA]^-$, 351.0552 $[2GlcA-H]^-$
S_{GU}-13 [m]	14.13	u $[M-H-2GlcA]^-$	719.3635 $[M-H-GlcA]^-$, 351.0554 $[2GlcA-H]^-$
S_{GU}-14 [m]	14.17	d/e/f $[M-H-2GlcA-Ara/Xyl]^-$	777.4429 $[M-H-GlcA]^-$, 469.3299 $[M-H-2GlcA-Ara/Xyl]^-$
S_{GU}-15[C]	14.38	g $[M-H-2GlcA]^-$	647.3791 $[M-H-GlcA]^-$, 471.3448 $[M-H-2GlcA]^-$, 351.0563 $[2GlcA-H]^-$
S_{GU}-16 [m]	14.6	o $[M-H-2GlcA-Rha]^-$	951.4619 $[M-H-C_2H_4O_2]^-$, 599.3514 $[M-H-C_2H_4O_2-2GlcA]^-$,497.1150 $[2GlcA·Rha-H]^-$,467.3169 $[M-H-C_2H_4O-2GlcA-Rha]^-$

S_{GU}-17[C]	15	j/k [M-H-2GlcA]$^-$	659.3494 [M-H-GlcA]$^-$, 483.3130 [M-H-2GlcA]$^-$
S_{GU}-18[C]	15.01	l [M-H-2GlcA]$^-$	497.2903 [M-H-2GlcA]$^-$
S_{GU}-19[m]	15.16	j/k [M-H-2GlcA]$^-$	659.3494 [M-H-GlcA]$^-$, 483.3130 [M-H-2GlcA]$^-$, 351.0554 [2GlcA-H]$^-$
S_{GU}-20[C]	15.22	r [M-H-3GlcA]$^-$	849.3576 [M-H-GlcA]$^-$, 497.1150 [2GlcA·Rha-H]$^-$, 351.0554 [2GlcA-H]$^-$
S_{GU}-21*[C]	15.31	e [M-H-2GlcA-Glc]$^-$	821.3964 [M-H-Glc]$^-$, 645.3637 [M-H-GlcA-Glc]$^-$, 469.3326 [M-H-2GlcA-Glc]$^-$, 351.0565 [2GlcA-H]$^-$
S_{GU}-22[m]	15.75	r [M-H-GlcA-Glc-C_2H_2O]$^-$	703.3715 [M-H-Glc]$^-$, 527.3386 [M-H-GlcA-Glc]$^-$, 485.3268 [M-H-GlcA-Glc-C_2H_2O]$^-$
S_{GU}-23*[C]	15.87	r [M-H-2GlcA]$^-$	703.3703 [M-H-GlcA]$^-$, 527.3374 [M-H-2GlcA]$^-$, 351.0554 [2GlcA-H]$^-$
S_{GU}-24[m]	16.03	m/n [M-H-2GlcA]$^-$	661.3601 [M-H-GlcA]$^-$, 485.3268 [M-H-2GlcA]$^-$, 351.0559 [2GlcA-H]$^-$
S_{GU}-25[m]	16.26	m/n [M-H-2Glc-Xyl]$^-$	617.3721 [M-H-2GlcA]$^-$, 485.3271[M-H-2Glc-Xyl]$^-$, 351.0558 [2GlcA-H]$^-$
S_{GU}-26[m]	16.3	r [M-H-GlcA-Glc]$^-$	703.3715 [M-H-Glc]$^-$, 527.3386 [M-H-GlcA-Glc]$^-$
S_{GU}-27[m]	16.37	m/n [M-H-2GlcA-Ara/Xyl]$^-$	617.3721 [M-H-2GlcA]$^-$, 485.3271 [M-H-2GlcA-Ara/Xyl]$^-$, 351.0558 [2GlcA-H]$^-$
S_{GU}-28[C]	16.53	m/n [M-H-GlcA-Glc]$^-$	661.3524 [M-H-Glc]$^-$, 485.3271 [M-H-GlcA-Glc]$^-$
S_{GU}-29[m]	16.83	a/b/c [M-H-2GlcA-Rha]$^-$	497.1002 [2GlcA·Rha-H]$^-$
S_{GU}-30*[C]	17.2	h [M-H-2GlcA]$^-$	643.3481 [M-H-GlcA]$^-$, 467.3163 [M-H-2GlcA]$^-$
S_{GU}-31*[C]	17.41	m [M-H-2GlcA]$^-$	661.3601 [M-H-GlcA]$^-$, 485.3268 [M-H-2GlcA]$^-$, 351.0559 [2GlcA-H]$^-$
S_{GU}-32[m]	17.51	a/b/c [M-H-2GlcA]$^-$	631.3805 [M-H-GlcA]$^-$, 455.3443 [M-H-2GlcA]$^-$
S_{GU}-33[C]	17.53	m/n [M-H-GlcA-Glc]$^-$	661.3524 [M-H-Glc]$^-$, 485.3271 [M-H-GlcA-Glc]$^-$
S_{GU}-34[m]	17.75	m/n [M-H-2GlcA]$^-$	661.3524 [M-H-Glc]$^-$, 485.3271 [M-H-GlcA-Glc]$^-$, 351.0559 [2GlcA-H]$^-$
S_{GU}-35[C]	17.78	h/i [M-H-2GlcA]$^-$	643.3510 [M-H-GlcA]$^-$, 467.467.3163 [M-H-2GlcA]$^-$, 351.0559 [2GlcA-H]$^-$
S_{GU}-36[m]	18	s/t [M-H-2GlcA]$^-$	687.3720 [M-H-GlcA]$^-$, 511.3531 [M-H-2GlcA]$^-$, 469.3323 [M-H-2GlcA-C_2H_2O]$^-$, 351.0559 [2GlcA-H]$^-$

S_{GU}-37 [m]	18.12	d/e/f [M-H-2GlcA-Xyl]$^-$	777.4111 [M-H-GlcA]$^-$, 469.3299 [M-H-2GlcA-Xyl]$^-$, 351.0542 [2GlcA-H]$^-$
S_{GU}-38 [m]	18.3	r [M-H-2GlcA]$^-$	703.3664 [M-H-GlcA]$^-$, 527.3397 [M-H-2GlcA]$^-$, 485.3320 [M-H-2GlcA-C_2H_2O]$^-$, 351.0565 [2GlcA-H]$^-$
S_{GU}-39 [m]	18.39	r [M-H-GlcA-Glc]$^-$	703.3715 [M-H-Glc]$^-$, 527.3386 [M-H-GlcA-Glc]$^-$, 485.3268 [M-H-2GlcA-C_2H_2O]$^-$
S_{GU}-40 [m]	18.48	441.3 [M-H-2GlcA]$^-$	617.3752 [M-H-GlcA]$^-$, 441.3302 [M-H-2GlcA]$^-$, 351.0577 [2GlcA-H]$^-$
S_{GU}-41 [m]	18.5	d/e/f [M-H-2GlcA-Rha]$^-$	821.3983 [M-H-Rha]$^-$, 645.3670 [M-H-Rha-GlcA]$^-$, 497.1002 [2GlcA-Rha-H]$^-$, 469.3323 [M-H-Rha-2GlcA]$^-$
S_{GU}-42[C]	18.66	m/n [M-H-2GlcA]$^-$	661.3601 [M-H-GlcA]$^-$, 485.3268 [M-H-2GlcA]$^-$, 351.0559 [2GlcA-H]$^-$
S_{GU}-43 [m]	18.75	o [M-H-2GlcA]$^-$	689.3898 [M-H-GlcA]$^-$, 513.3629 [M-H-2GlcA]$^-$
S_{GU}-44*[C]	19.01	e [M-H-2GlcA]$^-$	645.3670 [M-H-GlcA]$^-$, 469.3323 [M-H-2GlcA]$^-$, 351.0577 [2GlcA-H]$^-$
S_{GU}-45*[C]	19.11	e [M-H-2GlcA]$^-$	645.3670 [M-H-GlcA]$^-$, 469.3323 [M-H-2GlcA]$^-$, 351.0577 [2GlcA-H]$^-$
S_{GU}-46 [m]	19.23	d/e/f [M-H-GlcA-Glc]$^-$	631.3822 [M-H-GlcA]$^-$, 469.3297 [M-H-GlcA-Glc]$^-$
S_{GU}-47[C]	19.7	d/e/f [M-H-GlcA-Glc]$^-$	631.3822 [M-H-GlcA]$^-$, 469.3297 [M-H-GlcA-Glc]$^-$
S_{GU}-48[C]	20.01	a/b/c [M-H-2GlcA]$^-$	631.3822 [M-H-GlcA]$^-$, 469.3323 [M-H-2GlcA]$^-$, 351.0577 [2GlcA-H]$^-$
S_{GU}-49 [m]	20.14	a/b/c [M-H-2GlcA]$^-$	631.3822 [M-H-GlcA]$^-$, 469.3323 [M-H-2GlcA]$^-$, 351.0577 [2GlcA-H]$^-$
S_{GU}-50 [m]	20.39	d/e/f [M-H-2GlcA]-	645.3670 [M-H-GlcA]$^-$, 469.3323 [M-H-2GlcA]$^-$, 351.0577 [2GlcA-H]$^-$
S_{GU}-51[C]	20.79	d/e/f [M-H-2GlcA]$^-$	645.3670 [M-H-GlcA]$^-$, 469.3323 [M-H-2GlcA]$^-$, 351.0577 [2GlcA-H]$^-$
S_{GU}-52 [m]	21.1	d/e/f [M-H-GlcA-Glc]$^-$	601.3758 [M-H-GlcA]$^-$, 469.3323 [M-H-GlcA-Glc]$^-$
S_{GU}-53*[C]	21.49	g [M-H-2GlcA]$^-$	647.3791 [M-H-GlcA]$^-$, 471.3448 [M-H-2GlcA]$^-$, 351.0563 [2GlcA-H]$^-$
S_{GU}-54[C]	22.5	q [M-H-2GlcA]$^-$	629.3651 [M-H-GlcA]$^-$, 453.3201 [M-H-2GlcA]$^-$, 351.0561 [2GlcA-H]$^-$
S_{GU}-55*[C]	22.98	c [M-H-2GlcA]$^-$	631.3822 [M-H-GlcA]$^-$, 455.3515 [M-H-2GlcA]$^-$, 351.0562 [2GlcA-H]$^-$
S_{GU}-56 [m]	29.59	e [M-H]$^-$	425.3417 [M-H-CO_2]$^-$

S_{AM}-1^{C}	15.02	489.4 [M-H-2Glc-Xyl]$^-$	783.4547 [M-H-Glc]$^-$, 621.4390 [M-H-2Glc]$^-$, 489.3642 [M-H-2Glc-Xyl]$^-$
S_{AM}-2^{C}	16.8	489.4 [M-H-2Glc-Xyl]$^-$	783.4547 [M-H-Glc]$^-$, 621.4390 [M-H-2Glc]$^-$, 489.3642 [M-H-2Glc-Xyl]$^-$
S_{AM}-3*^{C}	17.9	489.4 [M-H-Glc-Xyl]$^-$	489.3587 [M-H-Glc-Xyl]$^-$
S_{AM}-4*^{C}	18.1	489.4 [M-H-Glc-Xyl]$^-$	621.4390 [M-H-Glc]$^-$, 489.3642 [M-H-Glc-Xyl]$^-$
S_{AM}-5*^{C}	19.77	489.4 [M-H-Ac-Glc-Xyl]$^-$	783.4547 [M-H-Ac]$^-$, 621.4390 [M-H-Ac-Glc]$^-$, 489.3642 [M-H-Ac-Glc-Xyl]$^-$
S_{AM}-6^{C}	20.89	489.4 [M-H-Ac-Xyl-Glc]$^-$	783.4547 [M-H-Ac]$^-$, 621.4390 [M-H-Ac-Glc]$^-$, 489.3642 [M-H-Ac-Glc-Xyl]$^-$
S_{AM}-7*^{C}	21.92	489.4 [M-H-Ac-Glc-Xyl]$^-$	783.4547 [M-H-Ac]$^-$, 621.4390 [M-H-Ac-Glc]$^-$, 489.3642 [M-H-Ac-Glc-Xyl]$^-$
S_{AM}-8^{C}	23.32	489.4 [M-H-2Ac-Glc-Xyl]$^-$	871.4691 [M+HCOO-Ac]$^-$, 783.4547 [M-H-2Ac]$^-$, 621.4390 [M-H-2Ac-Glc]$^-$, 489.3642 [M-H-2Ac-Glc-Xyl]$^-$
S_{AM}-9*^{C}	24.16	489.4 [M-H-2Ac-Glc-Xyl]$^-$	871.4691 [M+HCOO-Ac]$^-$, 783.4547 [M-H-2Ac]$^-$, 621.4390 [M-H-2Ac-Glc]$^-$, 489.3642 [M-H-2Ac-Glc-Xyl]$^-$
S_{AM}-10^{C}	25.29	489.4 [M-H-2Ac-Glc-Xyl]$^-$	871.4691 [M+HCOO-Ac]$^-$, 783.4547 [M-H-2Ac]$^-$, 621.4390 [M-H-2Ac-Glc]$^-$, 489.3642 [M-H-2Ac-Glc-Xyl]$^-$
S_{PG}-1^{m}	2.79	Q [M-H-3Glc]$^-$	817.5058 [M-H-Glc]$^-$, 799.4803 [M-H-Glc-H_2O]$^-$, 655.4559 [M-H-2Glc]$^-$, 637.4381 [M-H-2Glc-H_2O]$^-$, 493.3892 [M-H-3Glc]$^-$
S_{PG}-2^{m}	3.61	J/K/L [M-H-3Glc]$^-$	799.4847 [M-H-Glc]$^-$, 637.4314 [M-H-2Glc]$^-$, 475.3783 [M-H-3Glc]$^-$, 391.2837 [M-H-3Glc-C_6H_{12}]$^-$
S_{PG}-3^{m}	3.65	J/K/L [M-H-4Glc]-	799.4847 [M-H-Glc]$^-$, 637.4314 [M-H-2Glc]$^-$, 475.3783 [M-H-3Glc]$^-$, 391.2837 [M-H-3Glc-C_6H_{12}]$^-$
S_{PG}-4^{m}	3.86	Q [M-H-2Glc-Rha]$^-$	801.5052 [M-H-Glc]$^-$, 493.3945 [M-H-2Glc-Rha]$^-$
S_{PG}-5^{m}	5.22	M/N/O [M-H-2Glc-Rha]$^-$	815.4845 [M-H-Rha]$^-$, 653.4266 [M-H-Glc-Rha]$^-$, 491.3752 [M-H-2Glc-Rha]$^-$
S_{PG}-6^{m}	7.02	J/K/L [M-H-3Glc]$^-$	799.4847 [M-H-Glc]$^-$, 637.4314 [M-H-2Glc]$^-$, 475.3783 [M-H-3Glc]$^-$
S_{PG}-7^{m}	7.27	Q [M-H-Glc-Rha]$^-$	493.3892 [M-H-3Glc]$^-$

S_{PG}-8[C]	7.71	J/K/L $[M\text{-}H\text{-}2Glc\text{-}Xyl]^-$	799.4836 $[M\text{-}H\text{-}Xyl]^-$, 637.4317 $[M\text{-}H\text{-}Glc\text{-}Xyl]^-$, 475.3784 $[M\text{-}H\text{-}2Glc\text{-}Xyl]^-$, 391.2831 $[M\text{-}H\text{-}2Glc\text{-}Xyl\text{-}C_6H_{12}]^-$
S_{PG}-9[C]	7.74	J/K/L $[M\text{-}H\text{-}3Glc]^-$	799.4817 $[M\text{-}H\text{-}Glc]^-$, 637.4340 $[M\text{-}H\text{-}2Glc]^-$, 475.3737 $[M\text{-}H\text{-}3Glc]^-$
S_{PG}-10[C]	8.39	J/K/L $[M\text{-}H\text{-}2Glc\text{-}Xyl]^-$	799.4836 $[M\text{-}H\text{-}Xyl]^-$, 637.4317$[M\text{-}H\text{-}Glc\text{-}Xyl]^-$, 475.3784 $[M\text{-}H\text{-}2Glc\text{-}Xyl]^-$, 391.2831 $[M\text{-}H\text{-}2Glc\text{-}Xyl\text{-}C_6H_{12}]^-$
S_{PG}-11 [m]	8.56	R $[M\text{-}H\text{-}Glc\text{-}Xyl]^-$	639.4144 $[M\text{-}H\text{-}Glc]^-$, 507.2037 $[M\text{-}H\text{-}Glc\text{-}Xyl]^-$
S_{PG}-12 [m]	8.9	J/K/L $[M\text{-}H\text{-}3Glc]^-$	799.4817 $[M\text{-}H\text{-}Glc]^-$, 637.4340 $[M\text{-}H\text{-}2Glc]^-$, 475.3737 $[M\text{-}H\text{-}3Glc]^-$
S_{PG}-13 [m]	9.01	J/K/L $[M\text{-}H\text{-}3Glc]^-$	799.4817 $[M\text{-}H\text{-}Glc]^-$, 637.4340 $[M\text{-}H\text{-}2Glc]^-$, 475.3737 $[M\text{-}H\text{-}3Glc]^-$
S_{PG}-14*[C]	9.2	L $[M\text{-}H\text{-}2Glc]^-$	637.4314 $[M\text{-}H\text{-}Glc]^-$, 475.3783 $[M\text{-}H\text{-}2Glc]^-$, 391.2837 $[M\text{-}H\text{-}2Glc\text{-}C_6H_{12}]^-$
S_{PG}-15*[C]	9.37	L $[M\text{-}H\text{-}2Glc\text{-}Rha]^-$	783.4855 $[M\text{-}H\text{-}Glc]^-$, 637.4304 $[M\text{-}H\text{-}Glc\text{-}Rha]^-$, 475.3775 $[M\text{-}H\text{-}2Glc\text{-}Rha]^-$
S_{PG}-16[C]	9.4	J/K/L $[M\text{-}H\text{-}2Glc]^-$	637.4314 $[M\text{-}H\text{-}Glc]^-$, 475.3783 $[M\text{-}H\text{-}2Glc]^-$, 391.2837 $[M\text{-}H\text{-}2Glc\text{-}C_6H_{12}]^-$
S_{PG}-17 [m]	9.8	J/K/L $[M\text{-}H\text{-}2Glc]^-$	637.4314 $[M\text{-}H\text{-}Glc]^-$, 475.3783 $[M\text{-}H\text{-}2Glc]^-$, 391.2837 $[M\text{-}H\text{-}2Glc\text{-}C_6H_{12}]^-$
S_{PG}-18 [m]	10.12	J/K/L $[M\text{-}H\text{-}2Glc]^-$	637.4314 $[M\text{-}H\text{-}Glc]^-$, 475.3783 $[M\text{-}H\text{-}2Glc]^-$, 391.2837 $[M\text{-}H\text{-}2Glc\text{-}C_6H_{12}]^-$
S_{PG}-19 [m]	10.86	Q $[M\text{-}H\text{-}2Glc\text{-}Rha]^-$	817.5058 $[M\text{-}H\text{-}Glc]^-$, 799.4803 $[M\text{-}H\text{-}Glc\text{-}H_2O]^-$, 655.4559 $[M\text{-}H\text{-}2Glc]^-$, 637.4381 $[M\text{-}H\text{-}2Glc\text{-}H_2O]^-$, 493.3875 $[M\text{-}H\text{-}2Glc\text{-}Rha]^-$, 475.3732 $[M\text{-}H\text{-}2Glc\text{-}Rha\text{-}H_2O]^-$, 375.7589 $[M\text{-}H\text{-}2Glc\text{-}Rha\text{-}H_2O\text{-}C_6H_{12}]^-$
S_{PG}-20 [m]	10.9	J/K/L $[M\text{-}H\text{-}Ac\text{-}2Glc\text{-}Rha]^-$	945.5423 $[M\text{-}H\text{-}Ac]^-$, 799.4847 $[M\text{-}H\text{-}Ac\text{-}Rha]^-$, 637.4314 $[M\text{-}H\text{-}Ac\text{-}Glc\text{-}Rha]^-$, 475.3783 $[M\text{-}H\text{-}Ac\text{-}2Glc\text{-}Rha]^-$, 391.2837 $[M\text{-}H\text{-}Ac\text{-}2Glc\text{-}Rha\text{-}C_6H_{12}]^-$
S_{PG}-21 [m]	11.5	J/K/L $[M\text{-}H\text{-}Glc\text{-}Rha\text{-}Xyl/Ara]^-$	753.4858 $[M\text{-}H\text{-}Glc]^-$, 607.4185 $[M\text{-}H\text{-}Glc\text{-}Rha]^-$, 475.3782

			[M-H-Glc-Rha-Xyl/Ara]$^-$
S_{PG}-22^{m}	11.63	J/K/L [M-H-Ac-2Glc-Rha]$^-$	945.5423 [M-H-Ac]$^-$, 799.4847 [M-H-Ac-Rha]$^-$, 637.4314 [M-H-Ac-Glc-Rha]$^-$, 475.3783 [M-H-Ac-2Glc-Rha]$^-$
S_{PG}-23^{m}	12.56	J/K/L [M-H-Glc-Rha-Xyl/Ara]$^-$	783.4884 [M-H-Xyl/Ara]$^-$, 637.4296 [M-H-Rha-Xyl/Ara]$^-$, 475.3782 [M-H-Glc-Rha-Xyl/Ara]$^-$
S_{PG}-24^{m}	12.72	P [M-H-4Glc]$^-$	963.5451 [M-H-4Glc]$^-$, 801.4897 [M-H-2Glc]$^-$, 639.4481 [M-H-3Glc]$^-$, 477.4033 [M-H-4Glc]$^-$
S_{PG}-25^{m}	13.09	J/K/L [M-H-Ac-2Glc-Rha]$^-$	945.5423 [M-H-Ac]$^-$, 783.4827 [M-H-Ac-Glc]$^-$, 637.4314 [M-H-Ac-Glc-Rha]$^-$, 475.3783 [M-H-Ac-2Glc-Rha]$^-$, 391.2837 [M-H-Ac-2Glc-Rha-C_6H_{12}]$^-$
S_{PG}-26^{m}	13.56	J/K/L [M-H-3Glc]$^-$	799.4817 [M-H-Glc]$^-$, 637.4340 [M-H-2Glc]$^-$, 475.3737 [M-H-3Glc]$^-$
S_{PG}-27^{C}	14	M/N/O [M-H-Glc-Xyl]$^-$	653.4175 [M-H-Xyl]$^-$, 491.3735 [M-H-Glc-Xyl]$^-$
S_{PG}-28^{m}	14.24	Q [M-H-2Glc-Rha]$^-$	801.5052 [M-H-Glc]$^-$, 493.3945 [M-H-2Glc-Rha]$^-$
S_{PG}-29*^{C}	14.46	L [M-H-2Glc]$^-$	637.4314 [M-H-Glc]$^-$, 475.3783 [M-H-2Glc]$^-$, 391.2837 [M-H-2Glc-C_6H_{12}]$^-$
S_{PG}-30*^{C}	14.57	O [M-H-Rha-Glc]$^-$	653.4260 [M-H-Rha]$^-$, 491.3739 [M-H-Rha-Glc]$^-$
S_{PG}-31^{C}	15.24	J/K/L [M-H-Xyl-Glc]$^-$	637.4302 [M-H-Xyl]$^-$, 475.3772 [M-H-Xyl-Glc]$^-$, 391.2845 [M-H-Xyl-Glc-C_6H_{12}]$^-$
S_{PG}-32^{C}	15.99	J/K/L [M-H-Glc]$^-$	475.3784 [M-H-Glc]$^-$, 391.2850 [M-H-Glc-C_6H_{12}]$^-$
S_{PG}-33*^{C}	16.08	L [M-H-Rha-Glc]$^-$	637.4318 [M-H-Rha]$^-$, 475.3784 [M-H-Rha-Glc]$^-$, 391.2850 [M-H-Rha-Glc-C_6H_{12}]$^-$
S_{PG}-34^{C}	16.38	J/K/L [M-H-Rha-Glc]$^-$	637.4318 [M-H-Rha]$^-$, 475.3784 [M-H-Rha-Glc]$^-$, 391.2850 [M-H-Rha-Glc-C_6H_{12}]$^-$
S_{PG}-35^{C}	16.5	J/K/L [M-H-Glc]$^-$	475.3784 [M-H-Glc]$^-$, 391.2850 [M-H-Glc-C_6H_{12}]$^-$
S_{PG}-36^{m}	16.77	G/H [M-H-2Xyl-3Glc]$^-$	1077.5848 [M-H-Xyl]$^-$,945.5418 [M-H-2Xyl]$^-$, 783.4890 [M-H-2Xyl-Glc]$^-$, 621.4382 [M-H-2Xyl-2Glc]$^-$, 459.3821

			[M-H-2Xyl-3Glc]$^{-}$
S_{PG}-37*^{C}	16.97	H [M-H-4Glc]$^{-}$	945.5386 [M-H-Glc]$^{-}$, 783.4864 [M-H-2Glc]$^{-}$, 621.4370 [M-H-3Glc]$^{-}$, 459.3810 [M-H-4Glc]$^{-}$
S_{PG}-38^{m}	17.32	G/H [M-H-Ac-4Glc]$^{-}$	1107.5953 [M-H-Ac]$^{-}$, 1089.5863 [M-H-Ac-H_2O]$^{-}$, 945.5384 [M-H-Ac-Glc]$^{-}$, 783.4899 [M-H-Ac-2Glc]$^{-}$, 621.4239 [M-H-Ac-3Glc]$^{-}$, 459.3872 [M-H-Ac-4Glc]$^{-}$
S_{PG}-39*^{C}	17.51	I [M-H-2Glc-GlcA]$^{-}$	793.4382 [M-H-Glc]$^{-}$, 631.3800 [M-H-2Glc]$^{-}$, 455.3562 [M-H-2Glc-GlcA]$^{-}$
S_{PG}-40*^{C}	17.51	H [M-H-Xyl-3Glc]$^{-}$	945.5418 [M-H-Xyl]$^{-}$, 783.4890 [M-H-Xyl-Glc]$^{-}$, 621.4382 [M-H-Xyl-2Glc]$^{-}$, 459.3821 [M-H-Xyl-3Glc]$^{-}$
S_{PG}-41^{C}	17.63	G/H [M-H-2Xyl-3Glc]$^{-}$	1077.5848 [M-H-Xyl]$^{-}$, 945.5418 [M-H-2Xyl]$^{-}$, 783.4890 [M-H-2Xyl-Glc]$^{-}$, 621.4382 [M-H-2Xyl-2Glc]$^{-}$, 459.3821 [M-H-2Xyl-3Glc]$^{-}$
S_{PG}-42^{C}	17.88	G/H [M-H-Ac-Xyl-3Glc]$^{-}$	945.5452 [M-H-Ac-Xyl]$^{-}$, 927.5374 [M-H-Ac-Xyl-H_2O]$^{-}$, 783.4885 [M-H-Ac-Xyl-Glc]$^{-}$, 765.4813 [M-H-Ac-Xyl-Glc-H_2O]$^{-}$, 621.4388 [M-H-Ac-Xyl-2Glc]$^{-}$, 603.4418 [M-H-Ac-Xyl-2Glc-H_2O]$^{-}$, 459.3835 [M-H-Ac-Xyl-3Glc]$^{-}$, 375.2939 [M-H-Ac-Xyl-3Glc-C_6H_{12}]$^{-}$
S_{PG}-43*^{C}	18.09	H [M-H-Xyl-3Glc]$^{-}$	945.5418 [M-H-Xyl]$^{-}$, 783.4890 [M-H-Xyl-Glc]$^{-}$, 621.4382 [M-H-Xyl-2Glc]$^{-}$, 459.3821 [M-H-Xyl-3Glc]$^{-}$
S_{PG}-44*^{C}	18.28	H [M-H-Xyl-3Glc]$^{-}$	945.5418 [M-H-Xyl]$^{-}$, 783.4890 [M-H-Xyl-Glc]$^{-}$, 621.4382 [M-H-Xyl-2Glc]$^{-}$, 459.3821 [M-H-Xyl-3Glc]$^{-}$
S_{PG}-45^{C}	18.37	G/H [M-H-Ac-Xyl-3Glc]$^{-}$	945.5452 [M-H-Ac-Xyl]$^{-}$, 927.5374 [M-H-Ac-Xyl-H_2O]$^{-}$, 783.4885 [M-H-Ac-Xyl-Glc]$^{-}$, 765.4813 [M-H-Ac-Xyl-Glc-H_2O]$^{-}$, 621.4388 [M-H-Ac-Xyl-2Glc]$^{-}$, 603.4418 [M-H-Ac-Xyl-2Glc-H_2O]$^{-}$, 459.3835

			[M-H-Ac-Xyl-3Glc]$^-$, 375.2939 [M-H-Ac-Xyl-3Glc-C_6H_{12}]$^-$
S_{PG}-46[C]	18.74	G/H [M-H-Ac-4Glc]$^-$	1107.5953 [M-H-Ac]$^-$, 1089.5863 [M-H-Ac-H_2O]$^-$, 945.5384 [M-H-Ac-Glc]$^-$, 783.4899 [M-H-Ac-2Glc]$^-$, 621.4239 [M-H-Ac-3Glc]$^-$, 459.3872 [M-H-Ac-4Glc]$^-$
S_{PG}-47[m]	19.1	I [M-H-Glc-GlcA]$^-$	631.3884 [M-H-Glc]$^-$, 455.3487 [M-H-Glc-GlcA]$^-$
S_{PG}-48*[C]	19.23	H [M-H-3Glc]$^-$	783.4855 [M-H-Glc]$^-$, 621.4365 [M-H-2Glc]$^-$, 459.3878 [M-H-3Glc]$^-$, 375.2877 [M-H-3Glc-C_6H_{12}]$^-$
S_{PG}-49[C]	19.25	G/H [M-H-Ac-Xyl-3Glc]$^-$	945.5452 [M-H-Ac-Xyl]$^-$, 927.5374 [M-H-Ac-Xyl-H_2O]$^-$, 783.4885 [M-H-Ac-Xyl-Glc]$^-$, 765.4813 [M-H-Ac-Xyl-Glc-H_2O]$^-$, 663.4493 [M-H-Xyl-2Glc]$^-$, 621.4388 [M-H-Ac-Xyl-2Glc]$^-$, 603.4418 [M-H-Ac-Xyl-2Glc-H_2O]$^-$, 459.3835 [M-H-Ac-Xyl-3Glc]$^-$, 375.2939 [M-H-Ac-Xyl-3Glc-C_6H_{12}]$^-$
S_{PG}-50[m]	19.53	G/H [M-H-Ac-3Glc]$^-$	783.4887 [M-H-Ac-Glc]$^-$, 621.4347 [M-H-Ac-2Glc]$^-$, 459.3852 [M-H-Ac-3Glc]$^-$
S_{PG}-51[m]	19.54	G/H [M-H-Malonyl-3Glc]$^-$	945.5403 [M-H-Malonyl]$^-$, 783.4855 [M-H-Malonyl-Glc]$^-$, 621.4365 [M-H-Malonyl-2Glc]$^-$, 459.3878 [M-H-Malonyl-3Glc]$^-$, 375.2877 [M-H-Malonyl-3Glc-C_6H_{12}]$^-$
S_{PG}-52*[C]	19.54	G/H [M-H-3Glc]$^-$	783.4855 [M-H-Glc]$^-$, 621.4365 [M-H-2Glc]$^-$, 459.3878 [M-H-3Glc]$^-$, 375.2877 [M-H-3Glc-C_6H_{12}]$^-$
S_{PG}-53[m]	19.73	G/H [M-H-Ac-3Glc]$^-$	783.621.4347 [M-H-Ac-Glc]$^-$, 459.3852 [M-H-Ac-3Glc]$^-$
S_{PG}-54[C]	19.77	G/H [M-H-3Glc]$^-$	783.4855 [M-H-Glc]$^-$, 621.4365 [M-H-2Glc]$^-$, 459.3878 [M-H-3Glc]$^-$, 375.2877 [M-H-3Glc-C_6H_{12}]$^-$
S_{PG}-55[C]	19.79	G/H [M-H-Ac-Xyl-3Glc]$^-$	945.5452 [M-H-Ac-Xyl]$^-$, 927.5374 [M-H-Ac-Xyl-H_2O]$^-$, 783.4885 [M-H-Ac-Xyl-Glc]$^-$, 765.4813 [M-H-Ac-Xyl-Glc-H_2O]$^-$, 663.4493

			[M-H-Xyl-2Glc]$^-$, 621.4388 [M-H-Ac-Xyl-2Glc]$^-$, 603.4418 [M-H-Ac-Xyl-2Glc-H_2O]$^-$, 459.3835 [M-H-Ac-Xyl-3Glc]$^-$, 375.2939 [M-H-Ac-Xyl-3Glc-C_6H_{12}]$^-$
S$_{PG}$-56^C	20.17	G/H [M-H-3Glc]$^-$	783.4855 [M-H-Glc]$^-$, 621.4365 [M-H-2Glc]$^-$, 459.3878 [M-H-3Glc]$^-$, 375.2877 [M-H-3Glc-C_6H_{12}]$^-$
S$_{PG}$-57^C	20.35	G/H [M-H-Ac-3Glc]$^-$	783.4849 [M-H-Ac-Glc]$^-$, 621.4347 [M-H-Ac-2Glc]$^-$, 459.3852 [M-H-Ac-3Glc]$^-$
S$_{PG}$-58^m	20.75	G/H [M-H-Xyl-2Glc]$^-$	783.4897 [M-H-Xyl]$^-$, 765.4748 [M-H-Xyl-H_2O]$^-$, 621.4368 [M-H-Xyl-Glc]$^-$, 459.3809 [M-H-Xyl-2Glc]$^-$, 375.2876 [M-H-Xyl-2Glc-C_6H_{12}]$^-$
S$_{PG}$-59^C	21.19	G/H [M-H-Ac-3Glc]$^-$	783.621.4347 [M-H-Ac-Glc]$^-$, 459.3852 [M-H-Ac-3Glc]$^-$
S$_{PG}$-60^m	21.25	D/E/F [M-H-Xyl-Glc]$^-$	619.4210 [M-H-Xyl]$^-$, 457.3684 [M-H-Xyl-Glc]$^-$
S$_{PG}$-61^m	21.75	D/E/F [M-H-Xyl-Glc]$^-$	619.4210 [M-H-Xyl]$^-$, 457.3684 [M-H-Xyl-Glc]$^-$
S$_{PG}$-62*^{C}	21.79	F [M-H-Rha-Glc]$^-$	619.4276 [M-H-Rha]$^-$, 457.3452 [M-H-Rha-Glc]$^-$
S$_{PG}$-63*^{C}	22.27	E [M-H-Rha-Glc]$^-$	619.4276 [M-H-Rha]$^-$, 457.3452 [M-H-Rha-Glc]$^-$
S$_{PG}$-64^C	23.04	G/H [M-H-2Glc]$^-$	621.4338 [M-H-Glc]$^-$, 459.3880 [M-H-2Glc]$^-$
S$_{PG}$-65^m	23.5	I [M-H-Glc-GlcA]$^-$	631.3884 [M-H-Glc]$^-$, 455.3487 [M-H-Glc-GlcA]$^-$
S$_{PG}$-66*^{C}	24.4	H [M-H-2Glc]$^-$	621.4338 [M-H-Glc]$^-$, 459.3880 [M-H-2Glc]$^-$
S$_{PG}$-67^C	24.72	G/H [M-H-2Glc]$^-$	621.4338 [M-H-Glc]$^-$, 459.3880 [M-H-2Glc]$^-$
S$_{PG}$-68*^{C}	27.55	C [M-H-2Glc]$^-$	603.3776 [M-H-Glc]$^-$, 441.3452 [M-H-2Glc]$^-$
S$_{PG}$-69*^{C}	27.88	A [M-H-2Glc]$^-$	603.3776 [M-H-Glc]$^-$, 441.3452 [M-H-2Glc]$^-$
S$_{PG}$-70^m	28	A/B/C [M-H-Ac-2Glc]$^-$	765.4600 [M-H-Ac]$^-$, 603.3768 [M-H-Ac-Glc]$^-$, 441.2103 [M-H-Ac-2Glc]$^-$
S$_{PG}$-71^m	28.2	G/H [M-H-Glc]$^-$	459.3880 [M-H-Glc]$^-$
S$_{PG}$-72^m	28.41	G/H [M-H-Glc]$^-$	459.3880 [M-H-Glc]$^-$
S$_{PG}$-73^m	29.57	A/B/C [M-H-Ac-2Glc]$^-$	765.4600 [M-H-Ac]$^-$, 603.3768

			[M-H-Ac-Glc]$^-$, 441.2103 [M-H-Ac-2Glc]$^-$
F_{AM}-8[m]	6.4	FA-2 [M-H-2Glc]$^-$	433.1382 [M-H-Glc]$^-$, 271.0624 [M-H-2Glc]$^-$, 151.0033 [M-H-2Glc-C_8H_8O]$^-$, 119.0527 [M-H-2Glc-$C_7H_4O_4$]$^-$
F_{AM}-13[C]	7.46	FA-10 [M-H-Glc]$^-$	299.0550 [M-H-Glc]$^-$, 284.0317 [M-H-Glc]$^-$, 135.0079 [M-H-Glc-$C_9H_8O_3$]$^-$
F_{AM}-36[m]	10	FA-12 [M-H-Api-Glc]$^-$	463.1601 [M-H-Api]$^-$, 301.0707 [M-H-Api-Glc]$^-$
F_{AM}-38[m]	10.82	FA-5 [M-H-Glc]$^-$	269.0446 [M-H-Glc]$^-$
F_{AM}-40[m]	11.21	FA-10 [M-H-Glc]$^-$	299.0550 [M-H-Glc]$^-$, 284.0317 [M-H-Glc-CH_3]$^-$, 135.0079 [M-H-Glc-CH_3-$C_8H_5O_3$]$^-$
F_{AM}-41[m]	11.25	FA-7 [M-H-Glc]$^-$	283.0610 [M-H-Glc]$^-$, 268.0359 [M-H-Glc-CH_3]$^-$
F_{AM}-43[m]	11.44	FA-12 [M-H-2Glc]$^-$	463.1591 [M-H-Glc]$^-$, 301.0704 [M-H-2Glc]$^-$, 271.0627 [M-H-2Glc-2CH_3]$^-$
F_{AM}-47[m]	11.6	FA-8 [M-H-Glc]$^-$	285.0752 [M-H-Glc]$^-$
F_{AM}-49[m]	12.1	FA-12 [M-H-Glc]$^-$	301.1099 [M-H-Glc]$^-$, 271.0624 [M-H-Glc-2CH_3]$^-$
F_{AM}-50[m]	12.28	FA-12 [M-H-Api]$^-$	403.1396 [M-H-CH_2O]$^-$, 373.1224 [M-H-2CH_2O]$^-$, 301.1099 [M-H-Api]$^-$, 283.0561 [M-H-Api-H_2O]$^-$
F_{AM}-54[m]	12.8	FA-3 [M-H]$^-$	135.0085 [M-H-C_8H_6O]$^-$
F_{AM}-60[m]	13.3	FA-12 [M-H-Glc]$^-$	301.1099 [M-H-Glc]$^-$, 271.0624 [M-H-Glc-CH_3]$^-$
F_{AM}-62[m]	13.6	FA-7 [M-H]$^-$	283.0607 [M-H]$^-$, 268.0450 [M-H-CH_3]$^-$
F_{AM}-64*[m]	13.7	FA-3 [M-H]$^-$	135.0085 [M-H-C_8H_6O]$^-$
F_{AM}-67[m]	13.91	FA-4 [M-H-Acetyl-Glc]$^-$	267.1 [M-H-Acetyl-Glc]$^-$, 252.0420 [M-H-Acetyl-Glc-CH_3]$^-$, 135.0101 [M-H-Acetyl-Glc-CH_3-C_8H_5O]$^-$
F_{AM}-68[m]	14.2	FA-7 [M-H]$^-$	268.0450 [M-H-CH_3]$^-$
F_{AM}-69[m]	14.22	FA-1 [M-H-2Glc]$^-$	255.0657 [M-H-2Glc]$^-$, 135.0081 [M-H-2Glc-C_8H_8O]$^-$, 119.0495 [M-H-2Glc-$C_7H_4O_3$]$^-$
F_{AM}-70[m]	14.23	FA-11 [M-H-Api-Glc]$^-$	299.0897 [M-H-Api-Glc]$^-$, 284.0686 [M-H-Api-Glc-CH_3]$^-$, 269.0417 [M-H-Api-Glc-2CH_3]$^-$
F_{AM}-71[m]	14.47	FA-11 [M-H-Api-Glc]$^-$	299.0897 [M-H-Api-Glc]$^-$, 284.0686 [M-H-Api-Glc-CH_3]$^-$, 269.0417 [M-H-Api-Glc-2CH_3]$^-$
F_{AM}-72[C]	15.07	FA-12 [M-H-Glc]$^-$	301.1099 [M-H-Glc]$^-$, 271.0624

			[M-H-Glc-2CH$_3$]$^-$
F$_{AM}$-75*^{C}	15.22	FA-7 [M-H]$^-$	135.0081 [M-H-C$_8$H$_8$O]$^-$, 119.0495 [M-H-C$_7$H$_4$O$_3$]$^-$
F$_{AM}$-76*^{C}	15.7	FA-12 [M-H-Glc]$^-$	301.1099 [M-H-Glc]$^-$, 271.0624 [M-H-Glc-2CH$_3$]$^-$
F$_{AM}$-77^{m}	17.87	FA-5 [M-H]$^-$	133.0286 [M-H-C$_7$H$_3$O$_3$]$^-$
F$_{AM}$-78^{m}	18.3	FA-10 [M-H]$^-$	284.0305 [M-H-CH$_3$]$^-$, 269.0441 [M-H-2CH$_3$]$^-$
F$_{AM}$-79^{m}	18.9	FA-11 [M-H]$^-$	284.0682 [M-H-CH$_3$]$^-$, 269.0434 [M-H-2CH$_3$]$^-$
F$_{AM}$-80^{m}	19.01	FA-11 [M-H]$^-$	284.0682 [M-H-CH$_3$]$^-$, 269.0434 [M-H-2CH$_3$]$^-$
F$_{AM}$-81^{C}	19.09	FA-3 [M-H]$^-$	135.0085 [M-H-C$_8$H$_6$O]$^-$
F$_{AM}$-82*^{C}	20.25	FA-4 [M-H]$^-$	252.0419 [M-H-CH$_3$]$^-$
F$_{CC}$-3^{C}	4.97	FA-9 [M-H]$^-$	137.0238 [M-H-C$_8$H$_8$O$_3$]$^-$, 109.0296 [M-H-C$_9$H$_8$O$_4$]$^-$
F$_{CC}$-9^{C}	6.65	FA-9 [M-H]$^-$	137.0238 [M-H-C$_8$H$_8$O$_3$]$^-$, 109.0296 [M-H-C$_9$H$_8$O$_4$]$^-$
F$_{CC}$-11^{m}	6.69	FA-9 [M-H]$^-$	137.0238 [M-H-C$_8$H$_8$O$_3$]$^-$, 109.0296 [M-H-C$_9$H$_8$O$_4$]$^-$
F$_{GU,AM}$-25*^{C}	8.95	FA-7 [M-H-Glc]$^-$	283.0607 [M-H-Glc]$^-$, 268.0450 [M-H-Glc-CH$_3$]$^-$
F$_{GU,AM}$-51^{m}	12.3	FA-4 [M-H-Api-Glc]$^-$	267.0661 [M-H-Api-Glc]$^-$, 252.0420 [M-H-Api-Glc-CH$_3$]$^-$, 135.0101 [M-H-Api-Glc-CH$_3$-C$_8$H$_5$O]$^-$
F$_{GU,AM}$-55^{m}	13	FA-4 [M-H-Api-Glc]$^-$	267.0661 [M-H-Api-Glc]$^-$, 252.0420 [M-H-Api-Glc-CH$_3$]$^-$, 135.0101 [M-H-Api-Glc-CH$_3$-C$_8$H$_5$O]$^-$
F$_{GU}$-2^{m}	3.95	FA-1 [M-H-C$_4$H$_2$O$_2$-2Glc]$^-$	579.1732 [M-H-C$_4$H$_2$O$_2$]$^-$, 417.1187 [M-H-C$_4$H$_2$O$_2$-Glc]$^-$, 255.0657 [M-H-C$_4$H$_2$O$_2$-2Glc]$^-$, 135.0081 [M-H-C$_4$H$_2$O$_2$-2Glc-C$_8$H$_8$O]$^-$, 119.0495 [M-H-C$_4$H$_2$O$_2$-2Glc-C$_7$H$_4$O$_3$]$^-$
F$_{GU}$-4^{m}	5.7	FA-1 [M-H-C$_4$H$_2$O$_2$-Glc]$^-$	417.1187 [M-H-C$_4$H$_2$O$_2$]$^-$, 255.0657 [M-H-C$_4$H$_2$O$_2$-Glc]$^-$, 135.0081 [M-H-C$_4$H$_2$O$_2$-Glc-C$_8$H$_8$O]$^-$,119.0495 [M-H-C$_4$H$_2$O$_2$-Glc-C$_7$H$_4$O$_3$]$^-$
F$_{GU}$-5^{m}	5.87	FA-1 [M-H-C$_4$H$_2$O$_2$-Glc]$^-$	417.1187 [M-H-C$_4$H$_2$O$_2$]$^-$, 255.0657 [M-H-C$_4$H$_2$O$_2$-Glc]$^-$, 135.0081 [M-H-C$_4$H$_2$O$_2$-Glc-C$_8$H$_8$O]$^-$, 119.0495 [M-H-C$_4$H$_2$O$_2$-Glc-C$_7$H$_4$O$_3$]$^-$
F$_{GU}$-6^{m}	5.88	FA-1 [M-H-C$_4$H$_2$O$_2$-Api-Glc]$^-$	549.1642 [M-H-C$_4$H$_2$O$_2$]$^-$, 417.1187 [M-H-C$_4$H$_2$O$_2$-Api]$^-$, 255.0657 [M-H-C$_4$H$_2$O$_2$-Api-Glc]$^-$, 135.0081

			[M-H-$C_4H_2O_2$-Api-Glc-C_8H_8O]$^-$, 119.0495 [M-H-$C_4H_2O_2$-Api-Glc-$C_7H_4O_3$]$^-$
F_{GU}-7^{C}	6.18	FA-1 [M-H-2Glc]$^-$	417.1187 [M-H-Glc]$^-$, 255.0657 [M-H-2Glc]$^-$, 135.0081 [M-H-2Glc-C_8H_8O]$^-$, 119.0495 [M-H-2Glc-$C_7H_4O_3$]$^-$
F_{GU}-10^{m}	6.65	FA-1 [M-H-Api-2Glc]$^-$	549.1641 [M-H-Glc]$^-$, 417.1187 [M-H-Api-Glc]$^-$, 255.0657 [M-H-Api-2Glc]$^-$, 135.0081 [M-H-Api-2Glc-C_8H_8O]$^-$, 119.0495 [M-H-Api-2Glc-$C_7H_4O_3$]$^-$
F_{GU}-12^{C}	7.21	FA-5 [M-H-2Glc]$^-$	473.1127 [M-H-$C_4H_8O_4$]$^-$, 383.0768 [M-H-$C_4H_8O_4$-$C_3H_6O_3$]$^-$, 353.0674 [M-H-2$C_4H_8O_4$]$^-$, 325.0608 [M-H-2$C_4H_8O_4$-CO]$^-$
F_{GU}-14^{m}	7.66	FA-3 [M-H-Glc]$^-$	253.0512 [M-H-Glc]$^-$, 135.0110 [M-H-Glc-C_8H_6O]$^-$
F_{GU}-15^{m}	7.7	FA-2 [M-H-2Glc]$^-$	433.1127 [M-H-Glc]$^-$, 271.0624 [M-H-2Glc]$^-$, 151.0033 [M-H-2Glc-C_8H_8O]$^-$, 119.0527 [M-H-2Glc-$C_7H_4O_4$]$^-$
F_{GU}-16^{C}	8.1	FA-5 [M-H-Glc-Api]$^-$	503.1192 [M-H-$C_2H_4O_2$]$^-$, 443.098 [M-H-$C_4H_8O_4$]$^-$, 353.0674 [M-H-$C_4H_8O_4$-$C_3H_6O_3$]$^-$
F_{GU}-17^{C}	8.2	FA-1 [M-H-2Glc]$^-$	255.0657 [M-H-2Glc]$^-$, 135.0081 [M-H-2Glc-C_8H_8O]$^-$, 119.0495 [M-H-2Glc-$C_7H_4O_3$]$^-$
F_{GU}-18^{m}	8.34	FA-1 [M-H-Api-2Glc]$^-$	549.1609 [M-H-Glc]$^-$, 417.1187 [M-H-Api-Glc]$^-$, 255.0657 [M-H-Api-2Glc]$^-$, 135.0081 [M-H-Api-2Glc-C_8H_8O]$^-$, 119.0495 [M-H-Api-2Glc-$C_7H_4O_3$]$^-$
F_{GU}-19^{m}	8.41	FA-3 [M-H-Glc]$^-$	253.0512 [M-H-Glc]$^-$, 135.0110 [M-H-Glc-C_8H_6O]
F_{GU}-20^{m}	8.42	FA-3 [M-H-Glc-Api]$^-$	415.1027 [M-H-Api]$^-$, 253.0512 [M-H-Glc-Api]$^-$, 135.0110 [M-H-Glc-Api-C_8H_6O]$^-$
F_{GU}-21^{m}	8.64	FA-1 [M-H-Api-2Glc]$^-$	549.1609 [M-H-Glc]$^-$, 417.1187 [M-H-Api-Glc]$^-$, 255.0657 [M-H-Api-2Glc]$^-$, 135.0081 [M-H-Api-2Glc-C_8H_8O]$^-$, 119.0495 [M-H-Api-2Glc-$C_7H_4O_3$]$^-$

F_{GU}-22 [m]	8.75	FA-3 $[M-H-Glc-Api]^-$	415.1027 $[M-H-Api]^-$, 253.0512 $[M-H-Glc-Api]^-$, 135.0110 $[M-H-Glc-Api-C_8H_6O]^-$
F_{GU}-23[C]	8.75	FA-2 $[M-H-Glc]^-$	271.0606 $[M-H-Glc]^-$
F_{GU}-24[C]	8.82	FA-1 $[M-H-2Glc]^-$	255.0657 $[M-H-2Glc]^-$, 135.0081 $[M-H-2Glc-C_8H_8O]^-$, 119.0495 $[M-H-2Glc-C_7H_4O_3]^-$
F_{GU}-26[C]	8.96	FA-1 $[M-H-Glc]^-$	255.0668 $[M-H-Glc]^-$, 135.0092 $[M-H-Glc-C_8H_8O]^-$
F_{GU}-27[C]	9	FA-1 $[M-H-2Glc]^-$	417.1216 $[M-H-2Glc]^-$, 255.0657 $[M-H-2Glc]^-$, 135.0081 $[M-H-2Glc-C_8H_8O]^-$, 119.0495 $[M-H-2Glc-C_7H_4O_3]^-$
F_{GU}-28 [m]	9.01	FA-2 $[M-H-Glc]^-$	271.0624 $[M-H-Glc]^-$, 151.0033 $[M-H-Glc-C_8H_8O]^-$, 119.0527 $[M-H-Glc-C_7H_4O_4]^-$
F_{GU}-29[C]	9.02	FA-1 $[M-H-Api-Glc]^-$	417.1216 $[M-H-Api-Glc]^-$, 255.0657 $[M-H-Api-Glc]^-$, 135.0081 $[M-H-Api-Glc-C_8H_8O]^-$, 119.0495 $[M-H-Api-Glc-C_7H_4O_3]^-$
F_{GU}-30*[C]	9.11	FA-1 $[M-H-Glc]^-$	255.0657 $[M-H-Glc]^-$, 135.0081 $[M-H-Glc-C_8H_8O]^-$, 119.0495 $[M-H-Glc-C_7H_4O_3]^-$
F_{GU}-31*[C]	9.4	FA-1 $[M-H-Api-Glc]^-$	417.1216 $[M-H-Api-Glc]^-$, 255.0657 $[M-H-Api-Glc]^-$, 135.0081 $[M-H-Api-Glc-C_8H_8O]^-$, 119.0495 $[M-H-Api-Glc-C_7H_4O_3]^-$
F_{GU}-32 [m]	9.51	FA-8 $[M-H-Glc]^-$	285.0695 $[M-H-Glc]^-$
F_{GU}-33[C]	9.61	FA-5 $[M-H-Rha-Glc]^-$	473.1083 $[M-H-C_4H_8O_3]^-$, 457.1123 $[M-H-C_4H_8O_4]^-$, 383.0755 $[M-H-C_4H_8O_3-C_3H_6O_3]^-$, 353.0664 $[M-H-C_4H_8O_3-C_4H_8O_4]^-$
F_{GU}-34 [m]	9.7	FA-2 $[M-H-Api-Glc]^-$	433.1127 $[M-H-Api]^-$, 271.0609 $[M-H-Api-Glc]^-$, 243.0657 $[M-H-Api-Glc-CO]^-$
F_{GU}-35 [m]	9.7	FA-8 $[M-H-Glc]^-$	285.0695 $[M-H-Glc]^-$
F_{GU}-37 [m]	10.6	FA-1 $[M-H-Glc]^-$	255.0657 $[M-H-Glc]^-$, 135.0081 $[M-H-Api-Glc-C_8H_8O]^-$, 119.0495 $[M-H-Api-Glc-C_7H_4O_3]^-$
F_{GU}-39 [m]	11	FA-8 $[M-H-Glc]^-$	285.0695 $[M-H-Glc]^-$
F_{GU}-42 [m]	11.25	FA-8 $[M-H-Glc]^-$	285.0695 $[M-H-Glc]^-$
F_{GU}-44 [m]	11.48	FA-2 $[M-H-Api-Glc]^-$	433.1127 $[M-H-Api]^-$, 271.0624 $[M-H-Api-Glc]^-$, 151.0033 $[M-H-Api-Glc-C_8H_8O]^-$

F_{GU}-45[C]	11.48	FA-2 [M-H-Glc]$^-$	271.0624 [M-H-Glc]$^-$, 151.0033 [M-H-Glc-C_8H_8O]$^-$
F_{GU}-46 [m]	11.5	FA-1 [M-H-2Glc]$^-$	417.1216 [M-H-2Glc]$^-$, 255.0657 [M-H-2Glc]$^-$, 135.0081 [M-H-2Glc-C_8H_8O]$^-$, 119.0495 [M-H-2Glc-$C_7H_4O_3$]$^-$
F_{GU}-48 [m]	11.9	FA-1 [M-H-Api-Glc]$^-$	417.1216 [M-H-Api]$^-$, 255.0657 [M-H-Api-Glc]$^-$, 135.0081 [M-H-Api-Glc-C_8H_8O]$^-$, 119.0495 [M-H-Api-Glc-$C_7H_4O_3$]$^-$
F_{GU}-52 [m]	12.4	FA-6 [M-H-Glc]$^-$	269.0819 [M-H-Glc]$^-$
F_{GU}-53 [m]	12.75	FA-7 [M-H-Glc]$^-$	283.0610 [M-H-Glc]$^-$, 268.0359 [M-H-Glc-CH_3]$^-$, 135.0110 [M-H-Glc-CH_3-$C_8H_5O_2$]$^-$
F_{GU}-56 [m]	13	FA-1 [M-H-2Glc]$^-$	255.0657 [M-H-2Glc]$^-$, 135.0081 [M-H-2Glc-C_8H_8O]$^-$, 119.0495 [M-H-2Glc-$C_7H_4O_3$]$^-$
F_{GU}-57*[C]	13.1	FA-4 [M-H-Glc]$^-$	267.0621 [M-H-Glc]$^-$, 252.0420 [M-H-Glc-CH_3]$^-$, 135.0101 [M-H-Glc-CH_3-C_8H_5O]$^-$
F_{GU}-58 [m]	13.1	FA-1 [M-H-Ac-2Glc]$^-$	549.1609 [M-H-Ac]$^-$, 417.1216 [M-H-Ac-Glc]$^-$, 255.0657 [M-H-Ac-2Glc]$^-$, 135.0081 [M-H-Ac-2Glc-C_8H_8O]$^-$, 119.0495 [M-H-Ac-2Glc-$C_7H_4O_3$]$^-$
F_{GU}-59*[C]	13.21	FA-1 [M-H-Api-Glc]$^-$	417.1216 [M-H-Glc]$^-$, 255.0657 [M-H-Api-Glc]$^-$, 135.0081 [M-H-Api-Glc-C_8H_8O]$^-$, 119.0495 [M-H-Api-Glc-$C_7H_4O_3$]$^-$
F_{GU}-61[C]	13.5	FA-1 [M-H-Glc]$^-$	255.0657 [M-H-Glc]$^-$, 135.0081 [M-H-Glc-C_8H_8O]$^-$, 119.0495 [M-H-Glc-$C_7H_4O_3$]$^-$
F_{GU}-63 [m]	13.63	FA-1 [M-H-Ac-Glc]$^-$	417.1187 [M-H-Ac]$^-$, 255.0650 [M-H-Ac-Glc]$^-$, 135.0077 [M-H-Ac-Glc-C_8H_8O]$^-$
F_{GU}-65*[C]	13.81	FA-1 [M-H-Api-Glc]$^-$	417.1216 [M-H-Api]$^-$, 255.0657[M-H-Api-Glc]$^-$, 135.0081 [M-H-Api-Glc-C_8H_8O]$^-$, 119.0495 [M-H-Api-Glc-$C_7H_4O_3$]$^-$
F_{GU}-66*[C]	13.9	FA-1 [M-H]$^-$	135.0081 [M-H-C_8H_8O]$^-$, 119.0495 [M-H-$C_7H_4O_3$]$^-$
F_{GU}-73 [m]	15.13	FA-2 [M-H-Glc]$^-$	271.0624 [M-H-Glc]$^-$, 151.0033 [M-H-Glc-C_8H_8O]$^-$

F_{GU}-74 [m]	15.2	FA-1 [M-H-Rha-Api-Glc]$^-$	417.1216 [M-H-Api]$^-$, 255.0657[M-H-Api-Glc]$^-$, 135.0081 [M-H-Api-Glc-C_8H_8O]$^-$, 119.0495 [M-H-Api-Glc-$C_7H_4O_3$]$^-$
F_{GU}-79 [m]	18.56	FA-1 [M-H-Ac-Glc]$^-$	417.1557 [M-H-Ac]$^-$, 255.065055.1 [M-H-Ac-Glc]$^-$,135.0077 [M-H Ac-Glc-C_8H_8O]$^-$
F_{GU}-83*[C]	20.26	FA-1 [M-H]$^-$	135.0080 [M-H-C_8H_8O]$^-$,119.0493 [M-H-$C_7H_4O_3$]$^-$
P_{CC}-1[C]	4.98	FA-9	425.087 [M-H-$C_8H_8O_3$]$^-$,289.071 [M-H-$C_{15}H_{13}O_6$]$^-$
P_{CC}-2[C]	5.73	FA-9	425.087 [M-H-$C_8H_8O_3$]$^-$,289.071 [M-H-$C_{15}H_{13}O_6$]$^-$
P_{CC}-3[C]	6.14	FA-9	425.087 [M-H-$C_8H_8O_3$]$^-$,289.071 [M-H-$C_{15}H_{13}O_6$]$^-$
P_{CC}-4[C]	6.54	FA-9	425.087 [M-H-$C_8H_8O_3$]$^-$,289.071 [M-H-$C_{15}H_{13}O_6$]$^-$
P_{CC}-5[C]	9.27	FA-9	425.087 [M-H-$C_8H_8O_3$]$^-$,289.071 [M-H-$C_{15}H_{13}O_6$]$^-$
P_{CC}-6[C]	10.46	FA-9	713.151 [M-H-$C_8H_8O_3$]$^-$,577.135 [M-H-$C_{15}H_{13}O_6$]$^-$,425.087 [M-H-$C_{15}H_{13}O_6$-$C_8H_8O_3$]-,289.071 [M-H-$C_{15}H_{13}O_6$]$^-$
L_{CC}-1[C]	10.02		521.202 [M-H-Api]$^-$,359.149 [M-H-Api-Glc]$^-$,341.139 [M-H-Api-Glc-H_2O]$^-$,326.115 [M-H-Api-Glc-H_2O-CH_3]$^-$
L_{CC}-2[C]	10.05		521.202 [M-H-Api]$^-$,359.149 [M-H-Api-Glc]$^-$,341.139 [M-H-Api-Glc-H_2O]$^-$,326.115 [M-H-Api-Glc-H_2O-CH_3]$^-$
L_{CC}-3[C]	10.29		389.159 [M-H-Glc]$^-$,371.149 [M-H-Glc-H_2O]$^-$,341.105 [M-H-Glc-H_2O-2CH_3]$^-$
L_{CC}-4[C]	10.39		359.149 [M-H-Glc]$^-$,341.139 [M-H-Glc-H_2O]$^-$,326.115 [M-H-Glc-H_2O-CH_3]$^-$
D_{CC}-1[C]	3.04		381.1913 [M-H-Glc]$^-$
D_{CC}-2*[C]	6.05		347.1857 [M-H-H_2O]$^-$
D_{CC}-3*[C]	6.51		407.2070 [M-H-H_2O]$^-$
D_{CC}-4*[C]	14.4		389.1964 [M-H-H_2O]$^-$

Notes, *: the compound identified by comparison with the reference.

[C]: the compound identified by UNIFI.

[m]: the compound detected by the multiple screening modes of Qtrap-MS.

F: flavonoid; S:saponin; P: procyanidin; L: lignan; D: diterpene.

$_{AM}$: the compound originated from *A. membranceus*; $_{GU}$: the compound originated from *G. uralensis*; $_{PG}$: the compound originated from *P. ginseng*; and $_{CC}$: the compound originated from *C. cassia*.

Table S2 The ion transitions, corresponding t_R, and optimal DPs and CEs for 175 quantitated and semi-quantitated compounds.

Analyte	t_R (min)	Ion transition (*m/z*)	DP (V)	CE (eV)
Re1/Re2/Re3/20-glu-Rf/NotoginsenosideM/N/Vinaginsenoside R4	3.61	1007 > 961	-200	-33
Q-*O*-Glc-Glc-Glc	2.79	1025 > 979	-200	-33
Apigenin-6,8-di-*C*-*β*-D-glucopyranoside*	3.43	593 > 473	-150	-38
Re1/Re2/Re3/20-glu-Rf/NotoginsenosideM/N/Vinaginsenoside R4	3.65	1007 > 961	-200	-33
Neoliquiritin apioside	4.02	549 > 255	-164	-41
Isomer of liquiritin apioside	4.40	549 > 255	-164	-41
Calycosin-7-*O*-*β*-D-glucoside*	4.64	445 > 283	-84	-22
Liquiritin*	4.67	417 > 255	-101	-30
Liquiritin apioside*	4.77	549 > 255	-164	-41
Catechin or epicatechin	4.97	289 > 245	-125	-25
PAC B-type dimer	4.98	577 > 407	-135	-25
Gypenoside Gc7	5.22	1007 > 961	-200	-33
PAC B-type dimer	5.73	577 > 407	-135	-25
PAC B-type dimer	6.14	577 > 407	-135	-25
Isomer of liquiritigenin-*O*-diglucoside	6.18	579 > 255	-135	-25
PAC B-type dimer	6.54	577 > 407	-135	-25
Isomer of calycosin	6.60	283 > 268	-90	-27
Catechin or Epicatechin	6.65	289 > 245	-125	-25
Catechin or Epicatechin	6.69	289 > 245	-125	-25
Re1/Re2/Re3/20-glu-Rf/NotoginsenosideM/N/Vinaginsenoside R4	7.02	1007 > 961	-200	-33
Isoliquiritin apioside*	7.06	549 > 255	-164	-41
Ginsenoside Rg1*	7.10	845 > 799	-150	-33
Ginsenoside Re*	7.15	991 > 945	-200	-33
Isoliquiritin*	7.17	417 > 255	-101	-30
Isomer of calycosin,	7.20	283 > 268	-90	-27
Liquiritigenin*	7.26	255 > 135	-80	-24
Ginsenoside Rf2	7.27	847 > 801	-150	-33
Ononin*	7.28	475 > 267	-100	-21
Isomer of ginsenoside Rg1	7.40	845 > 799	-150	-33
Isomer of 5'-hydroxy-4'-methoxyisoflavone-3'-*β*-D-glucoside	7.46	461 > 299	-125	-25
Re4/Notoginsenoside R1	7.71	977 > 931	-150	-33
Re1/Re2/Re3/20-glu-Rf/NotoginsenosideM/N/Vinaginsenoside R4	7.74	1007 > 961	-200	-33
Isomer of ginsenoside Rg1	7.80	845 > 799	-150	-33
Licuraside	7.81	549 > 255	-164	-41
Calycosin*	8.11	283 > 268	-90	-27
Isomer of ginsenoside Rg1	8.12	845 > 799	-150	-33
Uralsaponin C*	8.19	823 > 351	-185	-58
Liquiritigenin-*O*-diglucoside	8.20	579 > 255	-135	-25

Re4/Notoginsenoside R1	8.39	977 > 931	-150	-33
Yunganoside G2	8.44	839 > 351	-185	-58
Floralginsenoside C	8.56	847 > 801	-150	-33
5-Hydroxy-liquiritin or 8-Hydroxy-liquiritin	8.75	433 > 271	-135	-25
Liquiritigenin-*O*-diglucoside	8.82	579 > 255	-135	-25
Uralsaponin F*	8.88	895 > 351	-185	-58
Re1/Re2/Re3/20-glu-Rf/NotoginsenosideM/N/Vinaginsenoside R4	8.90	1007 > 961	-200	-33
g-*O*-Rha-GlcA-GlcA	8.90	969 > 351	-185	-58
Neoliquiritin	8.96	417 > 255	-101	-30
Liquiritigenin-*O*-diglucoside	9.00	579 > 255	-135	-25
Re1/Re2/Re3/20-glu-Rf/NotoginsenosideM/N/Vinaginsenoside R4	9.01	1007 > 961	-200	-33
5-Hydroxy-liquiritin or 8-Hydroxy-liquiritin	9.01	433 > 271	-135	-25
Isomer of uralsaponin F	9.22	895 > 351	-185	-58
PAC B-type dimer	9.27	577 > 407	-135	-25
Licorice-saponin A3*	9.67	983 > 351	-185	-58
Ginsenoside Rf*	9.71	845 > 799	-150	-33
Uralsaponin U/Uralsaponin N	9.90	837 > 351	-185	-58
Isomer of uralsaponin C	9.92	823 > 351	-185	-58
22β-acetoxyglycyrrhizin*	9.93	879 > 351	-185	-58
24-Hydroxyl-licorice E2/Yunganoside M	10.26	835 > 351	-185	-58
24-Hydroxy-licorice-saponin A3	10.38	999 > 351	-185	-58
Macedonoside C	10.56	821 > 351	-185	-58
Ginsenoside Rg2*	10.59	829 > 783	-150	-33
Isomer of liquiritin	10.60	417 > 255	-101	-30
Ginsenoside Rh1*	10.60	683 > 637	-135	-33
d/e/f-*O*-Xyl(Ara)-GlcA-GlcA	10.64	953 > 469	-185	-58
22-Hydroxy-licorice-saponin G2	10.67	853 > 501	-185	-58
Ginsenoside Rb1*	10.75	1153 > 1107	-200	-23
5,7-Dihydroxyl-flavone-4'-*O*-glucoside	10.82	431 > 269	-135	-25
Isomer of ginsenoside Rh1	10.82	683 > 637	-135	-33
Vinaginsenoside R13	10.86	1025 > 979	-200	-33
Acetyl-ginsenoside Re	10.90	1033 > 987	-200	-33
Licorice-saponin E2*	10.98	819 > 351	-185	-58
Licorice-saponin H2*	10.99	821 > 351	-185	-58
Uralsapionin D	11.01	849 > 497	-185	-58
Ginsenoside Rc*	11.02	1123 > 1107	-200	-23
Isomer of licorice-saponin H2	11.04	821 > 351	-185	-58
Licorice-saponin G2*	11.04	837 > 351	-185	-58
Isoliquiritigenin*	11.06	255 > 135	-80	-24
Not identified	11.08	851 > 351	-185	-58
Ginsenoside Ro*	11.09	955 > 793	-200	-50
Isomer of 5'-hydroxy-4'-methoxyisoflavone-3'-β-D-glucoside	11.21	461 > 299	-125	-25
Isomer of licorice-saponin H2	11.24	821 > 351	-185	-58
Isomer of calycosin-7-*O*-β-D-glucoside	11.25	445 > 283	-84	-22

Ginsenoside Rb2*	11.31	1123 > 1107	-200	-23
Yunganoside E2	11.46	819 > 351	-185	-58
5-Hydroxy-liquiritin or 8-Hydroxy-liquiritin	11.48	433 > 151	-135	-25
Isomer of liquiritigenin-*O*-diglucoside	11.50	579 > 255	-135	-25
J/K/L-*O*-Xyl(Ara)-Rha-Glc	11.50	961 > 915	-150	-33
Formononetin*	11.51	267 > 252	-125	-25
Ginsenoside Rb3*	11.52	1123 > 1077	-200	-23
24-Hydroxyl-licorice E2	11.56	835 > 483	-185	-58
5-Hydroxy-7-methoxyflavanone-5-*O*-glucoside	11.60	447 > 285	-125	-25
Acetyl-ginsenoside Re	11.63	1033 > 987	-200	-33
Ginsenoside Rg3*	12.06	829 > 783	-150	-33
Uralsaponin E	12.17	835 > 483	-185	-58
Ginsenoside Rd*	12.25	991 > 945	-200	-33
Isomucronulatol-*O*-apioside	12.28	433 > 301	-135	-25
Isomer of uralsaponin C	12.38	823 > 351	-185	-58
Genistin	12.40	431 > 269	-135	-25
Uralsaponin M	12.51	879 > 351	-185	-58
22*β*-Acetoxyglycyrrhizic acid	12.52	865 > 485	-185	-58
Isomer of ginsenoside Rd	12.54	991 > 945	-200	-33
J/K/L-*O*-Glc-Rha-Xyl(Ara)	12.56	961 > 915	-150	-33
P-*O*-Glc-Glc-Glc-Glc	12.72	1171 > 1125	-200	-33
Isomer of calycosin-7-*O*-*β*-D-glucoside	12.75	445 > 283	-84	-22
G/H-*O*-Glc-Glc-Glc-Xyl(Ara)-Xyl(Ara)	12.77	1255 > 1029	-200	-33
Isomer of isoliquiritigenin-*O*-diglucoside	13.00	579 > 255	-135	-25
Isomer of ginsenoside Rg3	13.04	829 > 783	-150	-33
Uralsaponin U/Uralsaponin N	13.04	837 > 351	-185	-58
Acetyl-ginsenoside Re	13.09	1033 > 987	-200	-33
Astragaloside II*	13.10	871 > 825	-150	-36
Isomucronulatol-7-*O*-*β*-D-glucoside	13.30	463 > 301	-125	-25
Re1/2/3,20-glu-Rf,Notoginsenoside N	13.56	1007 > 961	-200	-33
Acetyl-liquiritin/isoliquiritin	13.63	459 > 255	-135	-25
Yunganoside I2/Licorice saponin B2	13.64	807 > 351	-185	-55
Uralsaponin U/Uralsaponin N	13.73	837 > 351	-185	-58
Isomer of ginsenoside Rd	13.77	991 > 945	-200	-33
Ginsenoside Ra1	13.78	1255 > 1209	-200	-50
22*β*-Acetoxy-licorice-saponin B2	13.95	865 > 527	-185	-58
22*β*-Acetoxyglycyrrhaldehyde	13.96	863 > 351	-185	-58
Isoastragaloside II*	13.97	871 > 825	-150	-36
M/N/O-*O*-Glc-Xyl(Ara)	14.00	831 > 785	-150	-33
Liquiritigenin/Isoliquiritigenin-*O*-diglucoside	14.22	579 > 255	-135	-25
Rhaoglycyrrhizin	14.30	967 > 469	-185	-58
3,9-Dimethoxy-pterocarpane-10-*O*-glucoside-apioside	14.47	593 > 299	-125	-35
Pseudoginsenoside F11	14.57	845 > 799	-150	-33
r-*O*-GlcA-Glc	14.62	865 > 485	-185	-58

Isomer of ginsenoside Rg3	14.72	829 > 783	-150	-33
Ginsenoside Rg6	14.79	811 > 765	-150	-33
Quinquenoside R1	14.81	1195 > 1149	-200	-33
Uralsaponin P	14.90	823 > 351	-185	-58
Astragaloside V	15.02	991 > 945	-200	-33
Glycyrrhizic acid*	15.06	821 > 351	-190	-56
Isomucronulatol-7-*O*-*β*-D-glucoside	15.07	463 > 301	-125	-25
5-Hydroxy-liquiritin or 8-Hydroxy-liquiritin	15.13	433 > 151	-135	-25
Gypenoside XVII	15.17	991 > 945	-200	-33
Notoginsenoside R2	15.24	815 > 769	-150	-33
Pseudoginsenoside Rc1	15.35	1033 > 987	-200	-33
Ginsenoside F4*	15.41	811 > 765	-150	-33
Ginsenoside Rk1	15.55	811 > 765	-150	-33
G/H-*O*-Glc-Glc-Xyl(Ara)	15.75	961 > 915	-150	-33
Glycyrflavoside C	15.78	807 > 351	-185	-55
Cyclogaleginoside D	15.89	871 > 825	-150	-36
Uralsaponin P	16.03	823 > 351	-185	-58
d/e/f-*O*-GlcA-Glc	16.10	777 > 627	-190	-56
Pseudoginsenoside Rc1	16.19	1033 > 987	-200	-33
Notoginsenoside T5	16.25	797 > 751	-150	-33
Astragaloside I	16.32	913 > 867	-150	-32
Isomer of ginsenoside Rg2	16.38	829 > 783	-150	-33
22*β*-Acetoxy-licorice-saponin B2	16.38	865 > 513	-185	-58
Chikusetsusaponin Iva/Zingibroside R1	16.50	839 > 793	-150	-33
Licorice saponin J2	16.63	823 > 351	-185	-58
Notoginsenoside T5	16.75	797 > 751	-150	-33
Astragaloside VI	16.80	991 > 945	-200	-33
Isoastragaloside I	17.16	913 > 867	-150	-32
Astragaloside IV*	17.25	829 > 783	-135	-33
Cyclosieversioside B	17.29	913 > 867	-150	-32
Ginsenoside Rh2	17.50	667 > 621	-150	-33
Licorice-saponin B2	17.55	807 > 351	-185	-58
Isomer of ginsenoside Rh2	17.82	667 > 621	-150	-33
Resokaempferol	17.87	269 > 133	-125	-25
Ginsenoside Rs2	17.88	1165 > 1119	-200	-33
Astragaloside III	17.90	829 > 783	-135	-33
Isomer of pratensein	18.30	299 > 284	-125	-25
Ginsenoside Rs2	18.37	1165 > 1119	-200	-33
Acetyl-Liquiritin/Isoliquiritin	18.56	459 > 255	-135	-25
Quinquenoside R1	18.74	1195 > 1149	-200	-33
3-Hydroxy-9,10-dimethoxy-pterocarpane	18.90	299 > 284	-125	-25
10-Hydroxy-3,9-dimethoxy-pterocarpane	19.01	299 > 284	-125	-25
Ginsenoside Rs2	19.25	1165 > 1119	-200	-33
Pseudoginsenoside Rc1	19.53	1033 > 987	-200	-33

Pseudoginsenoside Rc1	19.73	1033 > 987	-200	-33
Ginsenoside Rs2	19.79	1165 > 1119	-200	-33
Ginsenoside Rs4/Rs5	20.00	853 > 807	-150	-33
Ginsenoside Rg5*	20.13	811 > 765	-150	-33
Ginsenoside Rs4/Rs5	21.57	853 > 807	-150	-33

*: the compound quantitated with the reference.

Table S3 The linear regression data, limits of quantification (LOQs) and detection (LODs) for all targeted 36 analytes.

Analytes	t_R(min)	Regression equation	r^2	Linear range (μg/mL)	LOD (ng/mL)	LOQ (ng/mL)
Apigenin-6,8-di-*C*-*β*-D-glucopyranoside (**1**)	3.43	y=0.0001x+0.0244	0.9997	0.2-28.06	7.23	22.20
Calycosin-7-*O*-*β*-D-glucoside (**2**)	4.64	y=0.00002x+0.00035	0.9999	0.2-24.05	6.78	18.89
Liquiritin (**3**)	4.67	y=0.0002x+0.013	0.9997	0.03-14.6	0.87	2.80
Liquiritin aposide (**4**)	4.77	y=0.00001x+0.00042	0.9996	0.05-12.30	4.12	12.01
Isoliquiritin aposide (**5**)	7.06	y=0.00001x-0.00075	0.9998	0.2-12.07	5.78	25.00
Ginsenoside Rg1 (**6**)	7.10	y=0.0005x+0.0481	0.9991	0.02-13.00	4.12	12.01
Ginsenoside Re (**7**)	7.15	y=0.00001x+0.000673	0.9991	0.01-4.50	3.50	17.66
Isoliquiritin (**8**)	7.17	y=0.0005x+0.0125	0.9997	0.01-5.33	0.04	0.20
Liquiritigenin (**9**)	7.26	y=0.001x+0.0618	0.9991	0.1-12.00	0.31	0.75
Ononin (**10**)	7.28	y=0.0003x-0.0213	0.9995	0.03-6.88	0.45	1.34
Calycosin (**11**)	8.11	y=0.0025x+0.2172	0.9997	0.02-8.77	0.09	0.17
Uralsaponin C (**12**)	8.19	y=0.000047x-0.059422	0.9993	0.1-4.17	7.51	13.01
Uralsaponin F (**13**)	8.88	y=0.0007x-0.0826	0.9996	0.15-5.04	3.95	15.75
Licorice saponin A3 (**14**)	9.67	y=0.00004x-0.03274	0.999	0.78-25.14	4.09	9.80
Ginsenoside Rf (**15**)	9.71	y=0.0017x+0.0140	0.9993	0.01-3.26	0.87	3.18
22*β*-Acetoxyglycyrrhizin (**16**)	9.93	y=0.0006x-0.3618	0.9996	0.70-23.76	23.21	46.41
Ginsenoside Rg2 (**17**)	10.59	y=0.0008x+0.4031	0.9994	0.1-9.40	4.52	9.20
Ginsenoside Rh1 (**18**)	10.60	y=0.00005x+0.01752	0.9997	0.1-13.12	2.10	5.27
Ginsenoside Rb1 (**19**)	10.75	y=0.0001x+0.0076	0.999	0.02-8.0	4.21	15.73
Licorice saponin E2 (**20**)	10.98	y=0.0001x-0.2117	0.9995	0.13-16.63	4.78	30.12
Licorice saponin H2 (**21**)	10.99	y=0.0011x-0.0176	0.999	0.02-1.3	0.74	1.48
Ginsenoside Rc (**22**)	11.02	y=0.00003x+0.00566	0.9991	0.1-14.0	13.59	27.18
Licorice saponin G2 (**23**)	11.04	y=0.0006x-0.3835	0.9995	0.1-13.22	7.56	25.08
Isoliquiritigenin (**24**)	11.06	y=0.0003x-0.1192	0.999	0.02-4.45	3.21	8.64
Ginsenoside Ro (**25**)	11.09	y=0.00001x+0.0032	0.9993	0.14-19.0	10.34	37.18
Ginsenoside Rb2 (**26**)	11.31	y=0.0001x+0.0005	0.9994	0.05-7.07	6.90	55.25
Fermononetin (**27**)	11.34	y=0.0019x-0.0286	0.9998	0.01-3.35	0.21	0.65
Ginsenoside Rb3 (**28**)	11.52	y=0.0001x+0.0057	0.9999	0.10-10.75	3.80	8.10
Ginsenoside Rg3 (**29**)	12.06	y=0.0006x+0.0853	0.9993	0.06-16.57	8.82	32.25
Ginsenoside Rd (**30**)	12.25	y=0.0003x+0.0014	0.9996	0.20-2.34	2.28	4.56
Astragaloside II (**31**)	13.10	y=0.0001x+0.0032	0.9997	0.03-8.24	8.05	32.21
Isoastragaloside II (**32**)	13.97	y=0.00001x+0.00129	0.9992	0.05-6.39	5.65	24.96
Glycyrrhizinic acid (**33**)	15.06	y=0.0007x-1.469	0.999	0.077-4.96	2.68	9.68
Ginsenoside F4 (**34**)	15.41	y=0.0001x-0.016	0.9995	0.05-13.01	2.14	9.06
Astragaloside IV (**35**)	17.25	y=0.0005x+0.0052	0.9998	0.02-2.21	2.15	8.61
Ginsenoside Rg5 (**36**)	20.13	y=0.0004x+0.013	0.9996	0.01-7.55	1.21	4.75

Table S4 The inter-day, intra-day precision, recovery performance, repeatability, and stability validation data for all monitored analytes (n = 6).

Analytes	Intra-day RSD (%)	Inter-day RSD (%)[a]	Recovery (%)	RSD (%)	Repeatability RSD (%)	Stability RSD (%)
Apigenin-6,8-di-*C*-*β*-D-glucopyranoside (**1**)	3.73	4.20	100.82	10.29	4.40	3.29
Calycosin-7-*O*-*β*-D-glucoside (**2**)	4.07	4.96	106.86	8.51	2.99	4.22
Liquiritin (**3**)	4.16	4.62	100.84	8.54	4.78	4.02
Liquiritin aposide (**4**)	2.66	3.88	100.72	7.46	4.10	4.76
Isoliquiritin aposide (**5**)	4.61	4.99	102.04	3.11	3.14	1.11
Ginsenoside Rg1 (**6**)	4.45	4.83	102.86	7.31	4.45	3.02
Ginsenoside Re (**7**)	2.98	4.30	101.27	7.26	2.83	4.11
Isoliquiritin (**8**)	3.76	3.95	101.29	11.06	2.70	4.16
Liquiritigenin (**9**)	1.70	3.45	103.49	5.01	2.55	2.55
Ononin (**10**)	3.16	4.61	99.91	6.28	4.10	4.06
Calycosin (**11**)	2.96	3.65	96.13	6.39	3.08	1.71
Uralsaponin C (**12**)	2.82	3.69	106.72	4.35	5.01	4.72
Uralsaponin F (**13**)	3.75	5.25	101.15	9.95	1.43	3.44
Licorice saponin A3 (**14**)	2.55	3.76	100.88	7.70	1.41	4.29
Ginsenoside Rf (**15**)	1.95	4.13	99.89	11.28	1.32	1.19
22*β*-Acetoxyglycyrrhizin (**16**)	1.64	3.07	99.59	9.84	2.04	3.01
Ginsenoside Rg2 (**17**)	3.57	4.57	98.58	12.65	4.98	3.71
Ginsenoside Rh1 (**18**)	3.44	3.49	90.68	6.27	2.16	2.92
Ginsenoside Rb1 (**19**)	3.54	4.72	102.72	4.86	2.92	1.71
Licorice saponin E2 (**20**)	0.96	2.37	108.92	4.67	4.23	4.23
Licorice saponin H2 (**21**)	3.89	3.68	103.82	8.32	3.52	3.47
Ginsenoside Rc (**22**)	1.97	3.72	108.15	12.07	2.68	2.96
Licorice saponin G2 (**23**)	3.11	3.33	107.20	9.95	3.55	4.03
Isoliquiritigenin (**24**)	3.13	3.25	106.87	4.41	3.27	4.44
Ginsenoside Ro (**25**)	2.72	4.14	103.77	11.20	2.40	4.41
Ginsenoside Rb2 (**26**)	3.41	4.93	103.68	3.74	4.26	4.63
Fermononetin (**27**)	1.48	1.39	100.37	5.30	3.15	4.77
Ginsenoside Rb3 (**28**)	4.56	4.73	109.91	10.01	3.93	3.93
Ginsenoside Rg3 (**29**)	2.86	3.58	103.21	13.21	4.42	4.42
Ginsenoside Rd (**30**)	3.48	3.97	99.50	8.97	4.04	4.94
Astragaloside II (**31**)	2.81	2.53	97.97	13.81	3.73	3.76
Isoastragaloside II (**32**)	2.65	4.60	98.78	12.31	3.81	3.81
Glycyrrhizinic acid (**33**)	3.12	2.88	97.08	10.86	1.85	3.14
Ginsenoside F4 (**34**)	2.85	3.50	102.31	9.78	4.67	4.67
Astragaloside IV (**35**)	2.74	4.70	97.01	7.79	2.78	3.86
Ginsenoside Rg5 (**36**)	3.25	4.07	107.21	3.67	4.54	4.24

[a]: n = 3
